# Unravelling Channel Structure–Diffusivity Relationships in Zeolite ZSM‐5 at the Single‐Molecule Level

**DOI:** 10.1002/anie.202114388

**Published:** 2021-12-02

**Authors:** Donglong Fu, J. J. Erik Maris, Katarina Stanciakova, Nikolaos Nikolopoulos, Onno van der Heijden, Laurens D. B. Mandemaker, Marijn E. Siemons, Desiree Salas Pastene, Lukas C. Kapitein, Freddy T. Rabouw, Florian Meirer, Bert M. Weckhuysen

**Affiliations:** ^1^ Inorganic Chemistry and Catalysis Debye Institute for Nanomaterials Science Utrecht University 3584 CG Utrecht The Netherlands; ^2^ Cell Biology Neurobiology and Biophysics Department of Biology Faculty of Science Utrecht University 3584 CG Utrecht The Netherlands

**Keywords:** Diffusion, Single-molecule studies, Thin films, Zeolites, ZSM-5

## Abstract

The development of improved zeolite materials for applications in separation and catalysis requires understanding of mass transport. Herein, diffusion of single molecules is tracked in the straight and sinusoidal channels of the industrially relevant ZSM‐5 zeolites using a combination of single‐molecule localization microscopy and uniformly oriented zeolite thin films. Distinct motion behaviors are observed in zeolite channels with the same geometry, suggesting heterogeneous guest–host interactions. Quantification of the diffusion heterogeneities in the sinusoidal and straight channels suggests that the geometry of zeolite channels dictates the mobility and motion behavior of the guest molecules, resulting in diffusion anisotropy. The study of hierarchical zeolites shows that the addition of secondary pore networks primarily enhances the diffusivity of sinusoidal zeolite channels, and thus alleviating the diffusion limitations of microporous zeolites.

## Graphical abstract

The microscopic channel structure–diffusivity relationships in zeolites were unraveled using a combination of single‐molecule localization microscopy and uniformly oriented zeolite thin films. New insights into the mechanism of the enhanced diffusion in hierarchical zeolites were obtained.

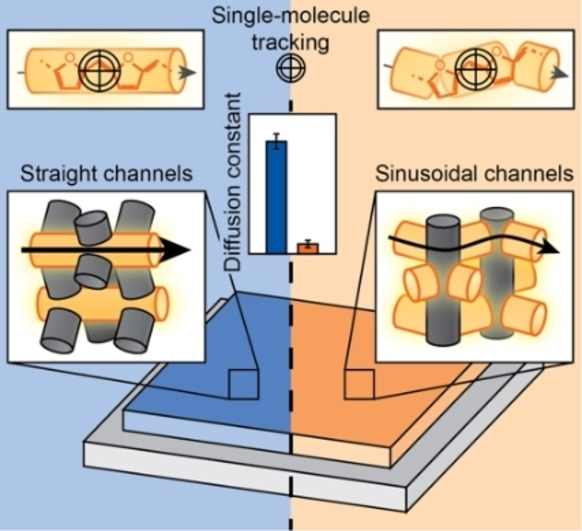

Zeolites are microporous aluminosilicates that have been utilized ubiquitously in industry for catalysis, selective adsorption and separation.[^1^, ^2^, ^3^] The pore/channel structure of zeolites strongly affects the overall performance by imposing diffusion limitations on the guest molecules.[^4^, ^5^, ^6^, ^7^] A prototypical case is the industrially heavily used ZSM‐5 zeolite (MFI framework, Figure 1) constituting interconnected sinusoidal (*a*‐direction) and straight (*b*‐direction)zeolite channels with distinct geometries and slightly different sizes. Superior molecular selectivity has been achieved both in catalysis and separation processes by carefully controlling the available channels to regulate diffusivities of target molecules.[^8^, ^9^, ^10^, ^11^] Moreover, the working lifetime of zeolites in catalysis has been dramatically improved by introducing secondary pore networks to overcome the intrinsic diffusion limit of the microchannels.[^5^, ^12^, ^13^, ^14^, ^15^] Understanding of the channel structure–diffusivity relationships is thus critical for rational improvement of zeolites’ performance and lifetime.

**Figure 1:**
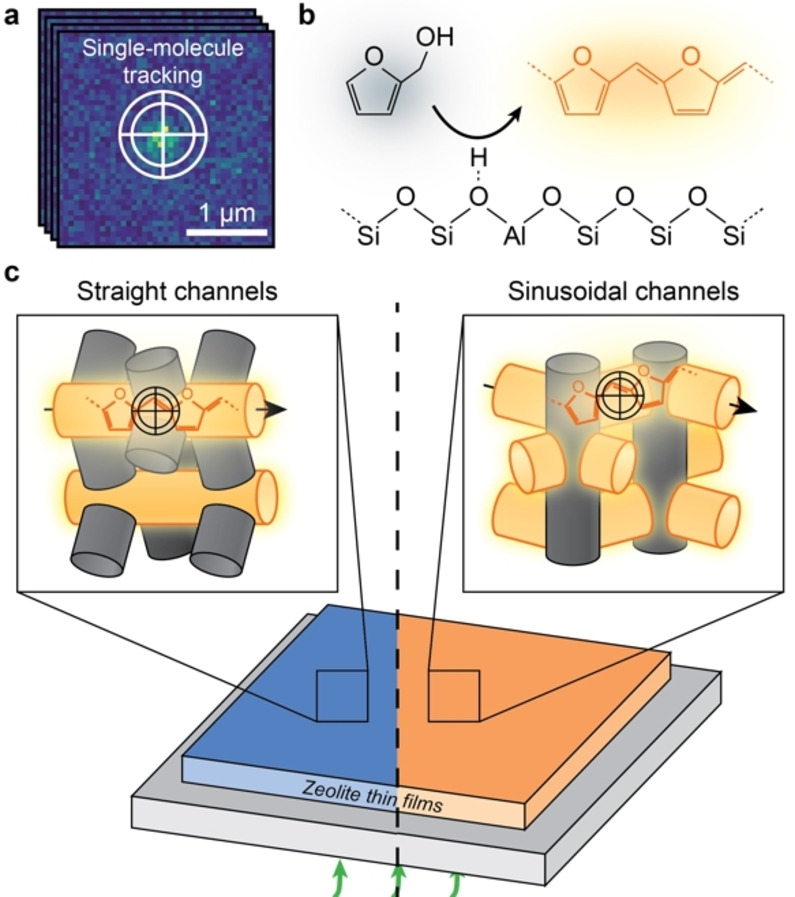
a) Illustration of the single‐molecule localization microscopy (SMLM) overcoming the diffraction limit by fitting the point spread function to the fluorescence signal. Trajectories are constructed by linking the positions of molecules against time. b) Formation of fluorescent products (orange) from the acid‐catalyzed oligomerization of furfuryl alcohol (black) on a Brønsted acid site. c) Schematic for probing the diffusivity of single molecules within single‐oriented zeolite channels over *a*‐oriented and *b*‐oriented zeolite ZSM‐5 thin films using SMLM with a 560 nm laser (green). Fluorescent products formed within the oriented channels parallel to the zeolite thin film (orange channels) are efficiently excited and tracked, whilst they are negligibly excited within oriented channels perpendicular to the thin film (grey channels), rendering them invisible to SMLM.

Great efforts have been dedicated in the past decades to the quantification of molecular mobility in zeolites,^[16]^ using a number of techniques, such as uptake measurements,[^17^, ^18^] pulsed field gradient nuclear magnetic resonance (PFG NMR) spectroscopy,[^6^, ^19^, ^20^, ^21^, ^22^] quasi‐elastic neutron scattering[^23^, ^24^, ^25^] and molecular simulations.[^26^, ^27^, ^28^] It was found that the zeolite channel geometry dictates the diffusivity. For instance, up to an order of magnitude differences in diffusivities were observed for the straight and sinusoidal zeolite channels of zeolite ZSM‐5.[^17^, ^20^, ^21^] However, a mechanistic understanding of this relationship remains elusive. Furthermore, the study of single crystals using microimaging techniques revealed intracrystalline heterogeneities of diffusivities in microporous materials.^[29]^ This highlights that molecular diffusion through zeolites is intrinsically heterogeneous, owing to their framework defects, heterogeneous distribution of adsorption sites, and channel connectivity.[^30^, ^31^, ^32^, ^33^, ^34^] Thus, to get a complete mechanistic understanding of channel structure–diffusivity relationships, it is of utmost importance to capture the diffusion heterogeneities at the single‐molecule level.

In this work, the diffusion heterogeneity in zeolite channels is quantified at the single‐molecule level in microporous and hierarchical zeolite ZSM‐5. As shown in Figure 1, this has been made possible by combining the recently developed, uniformly oriented zeolite thin films^[35]^ (Figure S1) with single‐molecule localization microscopy (SMLM, Figures 1a, S2–S3).^[36]^ This approach (Figure 1c) allows us to track diffusion of single molecules within the straight and sinusoidal zeolite channels over the *a*‐ and *b*‐oriented zeolite thin films, respectively. The maximum absorption and fluorescent emission of molecules is obtained when the electric field of the excitation light is parallel to the dipole moment of the entrapped molecules, which for FFA oligomers is along the trapped channels.[^37^, ^38^] Therefore, with a plane‐polarized laser, the molecules formed within the channels parallel to the substrate of the film will be efficiently excited and tracked. The single molecules were generated in situ from furfuryl alcohol (FFA) over the Brønsted acid sites in the zeolite thin films (Figures 1b,c, S4–S6).^[39]^ Time‐dependent density functional theory (TD‐DFT) computations indicate that the tetramer oligomers are specifically excited with a 560 nm laser (Figures S7,S8 with detailed discussions). It might be possible that one molecule can transfer from one channel type to the other. However, given the length of the molecule, we conjecture that it is unlikely that the molecules frequently change channel type. Altogether, the developed approach can be applied more broadly in a diagnostic manner to study the chemistry of porous materials at the level of single‐oriented channels.

An automatic detection algorithm for single‐molecule localization was used to reconstruct the trajectories of the fluorescent molecules (Figures S9 with detailed discussions). We demonstrated that the algorithm could successfully identify the full trajectory and extract reliable values of the diffusion coefficients via the mean squared displacement (MSD) curve (Figures S10–S13 with detailed discussions). A linear fit of the MSD curves of each trajectory in a representative oriented ZSM‐5 thin film results in a continuous range of diffusion coefficients spanning several orders of magnitude (Figures 2a and S14). This shows that guest molecules diffusing through macroscopically uniform zeolite channels have a very heterogeneous motion behavior. These results highlight the importance of tracking diffusion in zeolites at the single‐molecule level.

**Figure 2:**
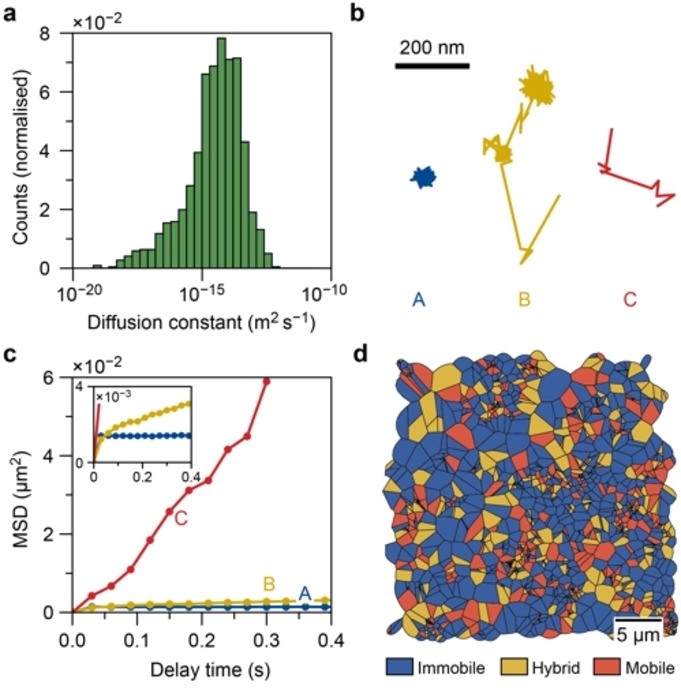
a) Histogram of the diffusion coefficients for all trajectories in the *a‐*oriented ZSM‐5 thin films with a logarithmic *x*‐axis. Fits resulted in a(n) (unphysical) negative diffusion constant are not shown. b) Diffusion patterns of three representative trajectories with immobile (A), hybrid (B), and mobile (C) motion types. c) The corresponding mean squared displacement curves of the trajectories in (b). d) Spatial map of the distribution of trajectories with different motion types. The area assigned to a trajectory is the Voronoi polygon located at the center of mass of the trajectory.

Further inspection of the individual trajectories shows three types of trajectories with distinct motion behaviors in the zeolite ZSM‐5 thin films (Figure 2b). Immobile molecules remain at or near a single location (type A), while mobile ones move almost constantly with large, up to 200 nm, steps between consecutive frames (type C). Only occasionally these molecules have short moments of impeded diffusion due to their interaction with the surroundings. The third group (type B) of molecules displays hybrid behavior and alternates between mobile and immobile periods with long segments of immobility. The different motion behavior is clearly reflected in the slope of the MSD curve, which is directly proportional to the diffusion constant (Figure 2c). Each trajectory is classified as either “mobile”, “hybrid”, or “immobile” based on the trajectory properties (Figure S15) following the previously developed approach.^[40]^ The observed two‐dimensional diffusion (Figure 2b) could be because the fluorophores travel through two or more different zeolite crystal domains oriented in the plane of the film. This is unlikely to occur frequently as the crystal domains are in the order of ca. 3 μm, therefore the diffusion constant would not be severely affected. The resulting spatial distribution (Figures 2d and S16) of the classified trajectories shows that the diffusion heterogeneity occurs randomly over the sample.

Next, the impact of channel geometry on diffusion heterogeneity was investigated to unravel the microscopic origin of diffusion anisotropy in zeolites. MSD analysis of individual trajectories (Figures 3a, b) reveals a continuous range of diffusion coefficients spanning several orders of magnitude for both channel orientations. We quantified the diffusion constant from a fit of the population‐averaged MSD curves (Figure S17). A large difference in the slope of these MSD curves (Figures 3c, d) of the mobile trajectories in the straight and sinusoidal zeolite channels is evident, while the immobile and hybrid trajectories’ MSD curves have a similar slope. The calculated diffusion coefficients (Figure S17) confirm that the mean diffusion coefficient of the mobile molecules is higher in the straight zeolite channels (2.64±0.24×10^−14^ m^2^ s^−1^) than in the sinusoidal zeolite channels (1.38±0.11×10^−14^ m^2^ s^−1^). The mean diffusion coefficient of the hybrid population is much lower than that of the mobile population because the hybrid population contains long immobile segments, while the diffusion coefficient of the immobile population approaches zero. The channel geometry also impacts the occurrence of each trajectory type (Figure 3e and Table S2). A two times larger fraction of mobile trajectories is observed in the straight zeolite channels (22 %) than in the sinusoidal analog (11 %). Meanwhile, fewer immobile trajectories are found in the straight zeolite channels than in sinusoidal zeolite channels. The effective diffusion coefficient (Figure 3f and Figure S19), calculated by considering the fraction and mobility of all types of trajectories (Figure S18), shows that the molecules move on average ten times faster in the straight zeolite channels (3.10±0.21×10^−15^ m^2^ s^−1^) than those in the sinusoidal zeolite channels (0.27±0.10×10^−15^ m^2^ s^−1^). The results of diffusion anisotropy are consistent with the results obtained by PFG NMR,[^19^, ^20^, ^21^, ^22^] validating the reliability of the developed method in this work. However, the latter fails to unlock the origin of diffusion anisotropy due to the ensemble nature of the technique. Collectively, these results demonstrate that the distinct channel geometries of the sinusoidal and straight channels of zeolite ZSM‐5 dictate the mobility and motion behavior of the molecules, resulting in diffusion anisotropy.

**Figure 3:**
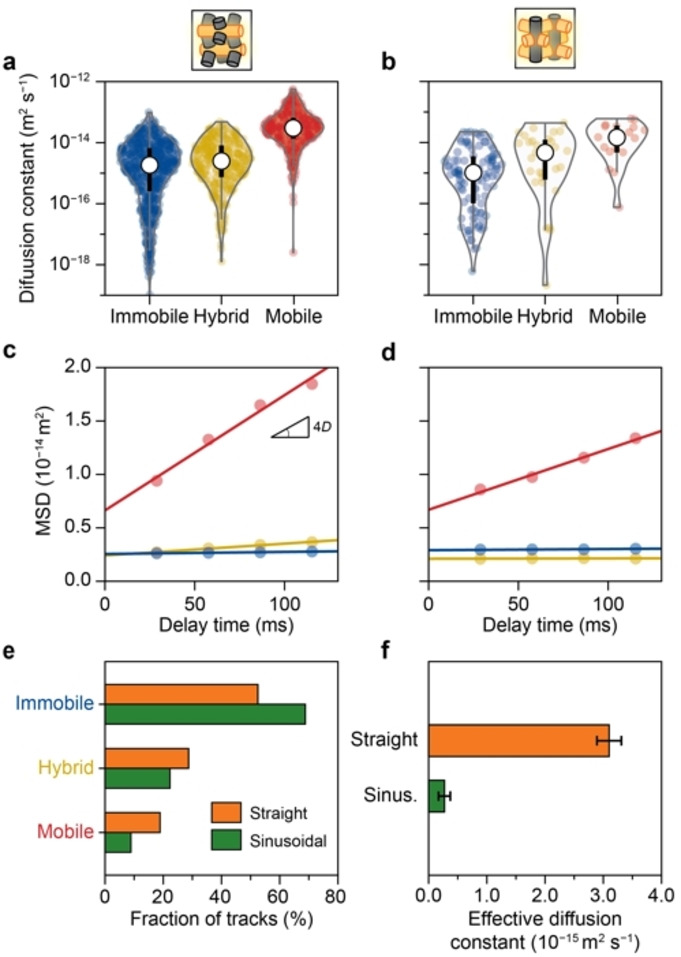
a,b) Violin plot of diffusion coefficients obtained via mean squared displacement (MSD) analysis of each individual trajectory in the (a) straight and (b) sinusoidal zeolite channels. The white dot and the bold black line indicate the median and first to third quartile of the distribution, respectively. c,d) The population‐averaged MSD curves of the “mobile”, “hybrid”, and “immobile” trajectories within the (c) straight and (d) sinusoidal zeolite channels. The circles and lines represent the experimental data and the linear fit of the MSD curve, respectively. e) The fraction of each type of trajectory within the straight (orange) and sinusoidal (green) zeolite channels. f) The effective diffusion coefficients of the straight (orange) and sinusoidal (green) zeolite channels. The error bars indicate the standard error. (See Supporting Information Videos 1–3).

Finally, the impact of the secondary pore networks on diffusivities within single‐oriented zeolite channels was investigated over hierarchical zeolite thin films (Figures 4a and S20–S21). Similar to the parent zeolite channels, a large span of diffusion coefficients for each population was observed in the hierarchical zeolite channels from individual MSD analysis (Figure S22). However, the diffusion anisotropy was significantly altered with both channel types possessing similar diffusion coefficients for the mobile population (≈2.5×10^−14^ m^2^ s^−1^, Figures 4b,c, and S17). Figure 4d reveals a higher fraction of mobile and hybrid trajectories as well as a lower fraction of immobile trajectories within the sinusoidal zeolite channels compared to the straight channels. These together results in a more than 1.5 times faster effective molecular diffusion within the sinusoidal zeolite channels (3.51±0.20×10^−15^ m^2^ s^−1^) than in their straight counterpart (2.20±0.24×10^−15^ m^2^ s^−1^, Figures 4e and S19).

**Figure 4:**
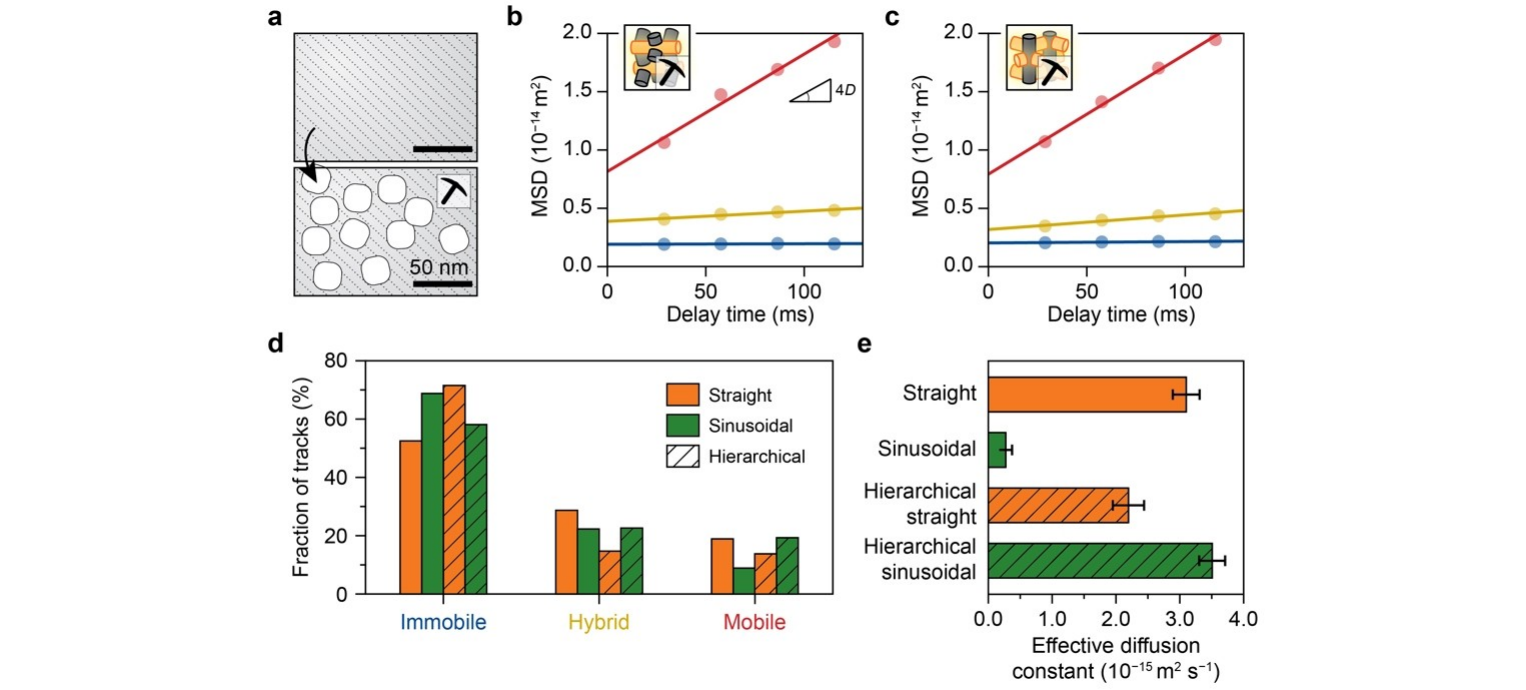
a) Schematic representation of the introduction of secondary pore networks into zeolites. b,c) The corresponding population‐averaged mean squared displacement (MSD) curves of the (b) straight and (c) sinusoidal zeolite channels after classification into “mobile”, “hybrid”, and “immobile” trajectories. The circles represent the experimental data, and the straight line is the fit of the MSD curve. d) The fraction of each type of trajectory within the straight (orange) and sinusoidal (green) zeolite channels with secondary porosity (hatched bars), in comparison with the corresponding parent zeolite channels. e) The effective diffusion coefficients for the straight (orange) and sinusoidal (green) zeolite channels with secondary porosity (hatched bars), in comparison with the corresponding parent zeolite channels. The error bars indicate the standard error. (See Supporting Information Videos 4 and 5).

Further, the change of diffusion heterogeneity in each channel orientation was quantified by comparing the parent and hierarchical zeolites. Surprisingly, while the sinusoidal zeolite channels show an order of magnitude increase in the effective diffusion coefficient with respect to the parent zeolite, a slight decrease was observed in the straight channels (Figure 4e). The increase in effective diffusivity in the sinusoidal zeolite channels is explained by a factor of two increase in both the diffusivity and fraction of the mobile molecules (Figures 4d and S17, Table S2). Interestingly, no apparent change in diffusivity (2.64±0.24 to 2.51±0.13×10^−14^ m^2^ s^−1^) was observed for the mobile molecules in the straight zeolite channels (Figures 4b and S17), while the fraction of immobile molecules increased at the expense of hybrid and mobile fractions (Figure 4d and Table S2), explaining the observed overall slight decrease in effective diffusivity. Previous works show that diffusivity in hierarchical zeolites was unaffected by steaming due to a lack of connectivity of the introduced meso pores.[^12^, ^31^] We suspect that the introduced secondary channels along the straight zeolite channels are disconnected. This conclusion is further corroborated by the above‐mentioned decline of the fraction of mobile trajectories from 22 % to 14 % in the straight channels (Figure 4d and Table S2), suggesting that some channels along the straight zeolite channels are likely blocked by the formation of extra‐framework silicon and aluminum. This was predicted by DFT,^[41]^ but it was never experimentally observed due to the limitations of current characterization techniques. Taken together, these results demonstrate that the addition of secondary pore networks primarily enhances the diffusivity of sinusoidal zeolite channels, and thus alleviating the diffusion limitations of microporous zeolites.

In summary, the channel structure–diffusivity relationships in zeolites were unraveled by capturing the diffusion heterogeneities at the single‐molecule level over single‐oriented zeolite channels. The results demonstrated that the distinct channel geometries dictate the mobility and motion behavior of the molecules, resulting in diffusion anisotropy. Further investigation of the diffusion heterogeneity in hierarchical zeolites provides direct insights on the working principle of the secondary pore networks on the enhancement of diffusivity. The results showed that the introduced secondary channels greatly promote the diffusivity in the sinusoidal zeolite channels and slightly suppresses the diffusivity in the straight analogue. Such knowledge is directly applicable to materials engineering using controlled channel structures thereby maximizing and/or suppressing the diffusion of reactant, product molecules as well as metal active sites.^[9]^

## Conflict of interest

The authors declare no conflict of interest.

## Supporting information

As a service to our authors and readers, this journal provides supporting information supplied by the authors. Such materials are peer reviewed and may be re‐organized for online delivery, but are not copy‐edited or typeset. Technical support issues arising from supporting information (other than missing files) should be addressed to the authors.

Supporting Information

Supporting Information

Supporting Information

Supporting Information

Supporting Information

Supporting Information
