# Supplementary material for: Unravelling Channel Structure–Diffusivity Relationships in Zeolite ZSM‐5 at the Single‐Molecule Level
Source: Angew Chem Int Ed Engl. 2021 Dec 2;61(5):e202114388. doi: 10.1002/anie.202114388 (PMC9299850; doi:10.1002/anie.202114388)
Supplement: Supplementary file 1 — Supporting Information [file ANIE-61-0-s004.pdf]

Angewandte Chemie
Eine Zeitschrift der Gesellschaft Deutscher Chemiker

Supporting Information

Unravelling Channel Structure–Diffusivity Relationships in Zeolite ZSM-5 at the Single-Molecule Level

D. Fu, J. J. E. Maris, K. Stanciakova, N. Nikolopoulos, O. van der Heijden, L. D. B. Mandemaker, M. E. Siemons, D. Salas Pastene, L. C. Kapitein, F. T. Rabouw, F. Meirer, B. M. Weckhuysen**

Table of Contents

Section S1. Experimental Details on Materials Synthesis 3

A. Chemicals 3

B. Detailed materials synthesis 3

Section S2. Physicochemical characterization 5

Section S3. Single-molecule localization 6

A. Details of experimental procedures 6

B. Data Analysis 6

C. Diffusion coefficient estimation 7

Section S4. Time-dependent density functional theory simulations 9

Section S5. Supplementary videos 10

Supplementary videos 1-2: 10

Supplementary video 3: 11

Supplementary video 4: 11

Supplementary video 5: 11

Section S6. Supplementary Figures S1-S22 12

Section S7. Supplementary Tables S1-S9 45

References 50

Section S1. Experimental Details on Materials Synthesis

A. Chemicals

Tetraethyl orthosilicate (TEOS, 98% (GC), Sigma Aldrich), tetrapropylammonium hydroxide (TPAOH, 1M aq., Alfa Aesar), bis(hexamethylene)triamine, 1-iodepropane, 2-butanone, anhydrous potassium carbonate, diethyl ether, ethyl acetate, potassium hydroxide (KOH, pellets, 85%, Alfa Aesar), aluminum sulfate hexadecahydrate ($Al_2(SO_4)_3 \cdot 18H_2O$, 98%, Sigma-Aldrich), ammonium nitrate (NH_4NO_3, >99%, Acros Organics), sodium silicate solution ($Na_2O(SiO_2)_x \cdot xH_2O$, ~ 10.6% Na_2O, ~ 26.5%SiO_2, Sigma-Aldrich), ethanol (anhydrous, absolute, Biosolve), sulfuric acid (95%, reagent grade, Fischer scientific), quartz plates (LSP Quartz B.V., 20 mm in diameter), 1,4-dioxane (99.8 %, Sigma-Aldrich), furfuryl alcohol (98 %, Sigma-Aldrich), branched polyethylenimine (PEI, Mw = 25000, Mn 10000, Sigma-Aldrich) were used as received.

B. Detailed materials synthesis

Structure-directing agent for seed crystals. Trimer-tetrapropylammonium cation (trimer-TPA^{3+}) was reported as the structure-directing agents (SDAs) for *a*-oriented crystals. The synthesis of trimer-TPA^{3+} was adapted from the procedure reported by Tsapatsis et al.,[1] where bis(hexamethylene)triamine was alkylated with 1-iodopropane. Typically, 450 mL of the solvent 2-butanone, an excess of 72.6 g of anhydrous potassium carbonate as HI and water scavenger, and 27.87 g of bis(hexamethylene)triamine were added to a three-neck 1L round bottom flask. A dropping funnel and a reflux condenser were connected to the flask. The reaction flask was located in a silicone oil bath on a stirring plate, flushed with nitrogen gas, and vented from the top of the condenser. The whole set-up was wrapped by aluminum foil to avoid iodide oxidation sensitive to light. The solution was gently heated and refluxed at 353 K under nitrogen atmosphere. Then 101 mL of 1-iodopropane was added dropwise with an addition funnel. The reaction was conducted overnight (~ 15 h) followed by filtration to remove the 2-butanone. The recovered solids contain the target product, trimer-$TPA^{3+}3I^-$ along with impurities, *i.e.,* KI and K_2CO_3. The solids were dissolved in 100 ml ethanol for several hours followed by filtration to obtain filtrate. An off-white solid, trimer-$TPA^{3+}3I^-$ with a small amount of impurity, was obtained after ethanol removal by rotary evaporation. The purification was done by adding in 250 mL of cold 2-butanone, after which equal volume of ethyl acetate was slowly added to precipitate out trimer-$TPA^{3+}3I^-$. After 10 h stirring the target product was recovered from the solution by filtration. A higher purity of the target product can be obtained

by repeating the latter part, *i.e.,* the cycle of ethanol extraction and recovery by diethyl ether and ethyl acetate. The purity was checked by ^{13}C NMR. TPAOH was used as received for the synthesis of *b*-oriented zeolite crystals. TPAOH was used as received for the synthesis of *b*-oriented crystals.

Synthesis of a-oriented and b-oriented silicalite-1 seed crystals. For the synthesis of the *a*-oriented silicalite-1 crystals with a leaf-like shape, a solution with a ratio of 6 TEOS: 0.75 timer-TPA^{3+}: 3.75 KOH: 1425 H_2O was prepared after stirring for 8 h. The solution was then poured into an autoclave through a filtration paper. The autoclave was placed in a rotational oven at 448 K for 24 h. The preparation of *b*-oriented silicalite-1 crystals followed Yoon's method.[2] The round, silicalite-1 crystals were synthesized from a solution composition of 6 TEOS:0.9 TPAOH:620 H_2O. The synthesis solution was prepared by adding TEOS to the solution containing TPAOH and H_2O. The mixture was transformed into a clear solution after stirring in a sealed liner for 24 h at room temperature. The clear solution was filtered with filter paper and charged into a Teflon-lined autoclave. The hydrothermal reaction was carried out in a rotation oven at 423 K for 12 h.

Manual assembly of an *a*-oriented and a *b*-oriented silicalite-1 monolayer. Typically, the quartz plates for the *a*-oriented and *b*-oriented zeolite ZSM-5 films were pre-coated with polyethylenimine (PEI) or pretreated with H_2O_2 (35 wt % aqueous solution), respectively. Then *ca*. 30 mg of *a*-oriented and *b*-oriented silicalite-1 crystals were put at the surface of quartz plates followed by pressing and rubbing using a finger with soft latex gloves. Subsequently, the loosely attached top layers were removed by gently wiping using glass wool for *ca.* 30 s. The *a*-oriented monolayers were calcined at 823 K overnight to remove the organic layers before secondary growth. The *b*-oriented monolayers were calcined for 2 h at 423 K to enhance the strength of the attachment.

Preparation of *a*-oriented and *b*-oriented Zeolite ZSM-5 films on quartz plates. The preparation of the *a*-oriented and *b*-oriented zeolite ZSM-5 films (thickness= *ca.* 800 nm) followed the same protocol reported by our group using the secondary growth method (see details in Supplementary Experimental Procedures).[3] The silicalite-1 monolayers supported on quartz plates were intergrown by the secondary growth method (SGM) at 448 K for 24 h. Typically, a solution comprising a required amount of $Al_2(SO_4)_3{\cdot}18\ H_2O$, 0.77 g H_2SO_4 (10 wt% aqueous solution) and 6.9 g of H_2O was added to a sodium silicate solution made by mixing 0.977 g of sodium silicate and 7.31 g H_2O as well as 0.32 g ethanol. After another 30 min of stirring at room temperature, a clear solution (Si/Al= 125) was obtained and was

transferred without filtration to a 20 mL Teflon-lined autoclave with the *a*-oriented and *b*-oriented crystals seeded substrates vertically placed at the bottom of the same autoclave. After the reaction, the autoclave was removed from the oven and quickly cooled to room temperature by immersing in cool H_2O. As-synthesized zeolite membranes on the substrates were carefully removed from the autoclave and washed with copious amounts of deionized H_2O and dried in air at 333 K.

After the synthesis, the films (Figure 1) were first treated with 0.2 M NH_4F solution for 2 h without stirring to remove the outermost amorphous silica layers that can block the channels of the membranes. Then the films were calcined at 823 K overnight with a ramp rate of 1.5 K/min under an airflow to remove the organic additive, *i.e.,* ethanol. The H-form sample was obtained by three times repeating the ion exchange of the calcined Na-type sample with a 1 M NH_4NO_3 solution (Acros Organic, 99+%) at 333 K overnight followed by calcination at 823 K for 6 h with a ramp rate of 1.5 K/min. The films with hierarchical pores were prepared using the fluoride etching method.[4] Typically, the as-synthesized zeolite films were put into a 40 wt% NH_4F aqueous solution and react at 333 K for 20 min under mechanical stirring. The etched products were thoroughly washed with distilled water after the fluoride treatment.

Section S2. Physicochemical characterization

The images of the zeolite monolayers and films were examined using a scanning electron microscope (SEM) from XL-30 (Philips) operating at an accelerating voltage of 5 kV. Before measurement, the surface of the materials was coated with a Pt layer of *ca.* 5 nm to avoid charging effects. X-ray diffraction (XRD) was used to confirm the orientation of the as-synthesized zeolite monolayers and films. The XRD patterns were collected using a Bruker D2 Phaser (2nd Gen) instrument using a cobalt radiation source, Co k_{α} = 1.789 Å. UV/Vis diffuse-reflectance spectroscopy (DRS) was applied to determine the absorption property of the zeolite ZSM-5 films after the oligomerization of furfuryl alcohol (FFA). The UV/Vis DRS spectra were collected using an AvaSpec 2048L spectrometer connected to a high-temperature UV/Vis optical fibre probe, which was used to collect spectra in reflection mode. Focused ion beam-scanning electron microscope (FIB-SEM) images were recorded on an FEI Helios NanoLab G3 UC (FEI Company) instrument. A protective layer of Pt was deposited on top of the region of interest before milling trenches on either side. Slices were milled perpendicular to the surface before SEM images were recorded (5 kV, 50 pA). Atomic force microscopy (AFM) micrographs were recorded using a Bruker Multimode 8 with a

n-type silicon tips with a hard diamond-like coating and aluminium background (having a resonance frequency of 160 kHz and a force constant of 5 N/m). The data was post-processed in Gwyddion.

Section S3. Single-molecule localization

A. Details of experimental procedures

The protocol for the fabrication and loading of the reaction cell used in the experiments is shown in Figure S2. Single-molecule localization experiments were performed using a custom-made fluorescence microscope setup (Figure S3). An inverted epi-fluorescence wide-field fluorescence microscope (Nikon Eclipse-TI) with a 100× oil immersion objective (NA=1.49) was used. A diode-pumped solid-state laser (Cobolt Jive-100, 560 nm) provided 54 mW to the sample. Fluorescence microscopy images were recorded as movies using an electron-multiplying charge-coupled device (EMCCD) camera (Andor iXon 897), after passing through a bandpass filter (Chroma, ZET560/10x), dichroic mirror (Chroma, ZT560rdc), long-pass filter (Chroma, ET575lp), quarter wave plate (Newport, 10RP44-1) and a 2.5× camera lens. Time-lapse videos were recorded with a resulting field of view of 32 × 32 μm^2 with 64 × 64 nm^2 per pixel (512 × 512 pixels), and a frame time of 30 ms. The reaction was performed in a Gene Frame cell (25 µL, 1 × 1 cm^2, Thermo Scientific, see details in Figure S2). The cell consists of a Gene Frame, which forms a sealed liquid microscopy cell after adhesion to a coverslip and microscope slide on each side. The oligomerization of FFA was performed on activated zeolite ZSM-5 films loaded on the top of the cover glass in the gene frame cell. The zeolite materials were exposed to furfuryl alcohol (FFA, 99%, Sigma Aldrich), previously diluted in 1,4-dioxane, to achieve the desired catalytic activity. The optimal concentration (see details in Table S1) of FFA for high-resolution imaging was determined in a series of concentration-dependent measurements. The sample was equilibrated for at least two hours and exposed to maximum laser power for 5 minutes (10.9 mW through objective) for fluorescence photobleaching before the measurement.

B. Data Analysis

The recorded SMLM microscopy movies were analysed with the DoM plugin (Detection of Molecules, https://github.com/ekatrukha/DoM_Utrecht) for ImageJ.[23] The localization of fluorescent events was done by independent classification of each frame into emissive spots and background. A list of initial emitter positions was determined with a sub-diffraction limited spatial resolution by fitting a 2-D Gaussian using the Levenberg−Marquardt least-squares algorithm. For trajectory analysis, molecules were

allowed to blink (*i.e.,* the molecule is fluorescing intermittently) for maximally 3 consecutive frames (90 ms) and travel 6 pixels (384 nm) between consecutive localizations (tracking result in Supplementary Movies 1–4). Only trajectories with more than four localizations were considered to ensure sufficient displacements per trajectory for MSD analysis and to remove unphysical trajectories originated from incorrect localizations. Trajectory classification, analysis and plotting were done in MATLAB (The Mathworks) using DiffusionLab, a software developed in our group for the classification and motion analysis of single-molecule trajectories. Previous work of our group has demonstrated the possibility of using a machine learning approach to group a large set of trajectories into populations with the same motion behaviour. The classification of the trajectories was done following a hierarchical decision tree built from a training set.[24] Inspired by this work, we manually built a decision tree to group the trajectories in this work in a rational manner. Motion analysis was performed with mean squared displacement (MSD) analysis of individual trajectories or a set of trajectories, *i.e.,* a population. The diffusion coefficient and localization error were obtained from a linear fit of the MSD curve. MSD analysis on individual trajectories was done including the first 25% of the delay times and at least three points. Only the first four points were used in the fit of the population MSD, because the number of trajectories contributing to the MSD was constant in this fit range.

C. Diffusion coefficient estimation

General notes for diffusion coefficient estimation. The free diffusion model in two dimensions were used for all motion types. We find that the population-averaged mean squared displacements are linear at short delay times (Figures 3c–d and 4b–c), indicating Brownian-type diffusion. One could expect that the channel geometry would give rise to one-dimensional diffusion. Nevertheless, a reason that we observed two-dimensional diffusion (Figure 2b) could be that the fluorophores travel through two or more different crystal domains with a different orientation in the observation plane. These domains are in the order of ~ 5 μm, therefore we don't expect this to occur very frequently and severely affecting the diffusion constant. We also fit the hybrid trajectories with a free diffusion model. A hybrid trajectory consists of mobile and immobile segments, due to transient trapping of the fluorophore. In two-dimensional diffusion, the population-averaged MSD curve is linear indicating normal diffusion. Here, the slope of the MSD curve is a weighted average of the mobile and immobile segments. In three-dimensional diffusion, the number of localizations per trajectory is heavily dependent on the length and number of immobile segments, because the fluorophore can diffuse out-of-focus during a mobile

segment. Thus, only long trajectories with many immobile displacements contribute at long delay times. This effect can give a plateau in the population-averaged MSD curve at long delay times, even though the fluorophores are not confined, which would give a similar effect. To prevent a bias in the measured slope of the MSD curve, the part of the MSD curve that considers displacements over longer delay times than the duration of the shortest trajectories must be excluded.

We determined the diffusion coefficient for individual trajectories and populations of trajectories with MSD analysis, as summarized in Table S2. To ensure sufficient displacements per trajectory for MSD analysis and remove artificial trajectories linked from random false localizations, we only considered trajectories with at least five localizations. Individual trajectories were analysed by fitting the mean squared displacement "MSD" as a function of lag time t,[25]

$$MSD = 4Dt + 4\sigma^2 \tag{7}$$

with D the diffusion coefficient, and σ the localization error. This relation considers two-dimensional normal diffusion with a fixed localization error. The lag time t is related to the number of frames over which the displacement spans n and the frame time Δt as $t = n\Delta t$. The least squares fit was weighed by the number of squared displacements that contributed to the mean, and only the first 25% of the trajectory (or at least three data points) were taken for the fit. Due to statistical dependence of the MSD values, data points at longer t are strongly correlated and reduce the accuracy of the MSD fit.[26] The optimum number of data points to include in the fit is only known for trajectories without blinking,[27] therefore we used a fixed fraction of 25%.

Diffusion coefficients from individual trajectories and all trajectories in a single population. Diffusion coefficients from individual trajectories were obtained by linear fitting of the MSD curves of each trajectory in a sample. The average motion of all trajectories in a single population via the “ensemble-averaged” (or population-averaged) MSD curve, which is constructed from the displacements of all trajectories. We fit equation (7) to the first four data points of the ensemble-averaged MSD curves. Only the first four data points were fit, because all trajectories had at least five localizations, from which we could extract displacements over up to at least four frames. At longer delay times, the MSD curve becomes irregular as not all trajectories in the population contribute to it anymore. These delay times were excluded from the fit.

Effective diffusion coefficient. Comparison with the previously reported ensemble diffusion coefficients, *e.g.,* obtained with PFG-NMR, requires the computation of an effective diffusion coefficient.[29] This is the weighted mean of the diffusion coefficient of each population following[29,30]

$$D_{\text{eff}} = p_{\text{mobile}} D_{\text{mobile}} + p_{\text{hybrid}} D_{\text{hybrid}}$$

with p_X and D_X the fraction of guest molecules and the diffusion in population X, respectively. As the diffusion coefficient of the immobile population is zero by definition, we leave this population out of the equation. We estimate weight p as the average fraction of molecules per frame, *i.e.* the average localization density of a population. We compute the molecules per frame and include the frames between the first and last localization when the molecule is off. We used the standard error of the mean to compute the error in p_X and the uncertainties were propagated following the well-known formulas.[31]

Section S4. Time-dependent density functional theory simulations

The static and dynamic adsorption maxima (Table S3) for the neutral and protonated oligomers were calculated using the time-dependent density functional theory (TD-DFT) simulations. The ground state structures of a series of neutral and charged furfuryl alcohol oligomers were obtained using DFT method as implemented in version 6.1 of CP2K software.[5] The gas-phase geometries were optimized using GAPW method[6] with CAM-B3LYP functional[7,8] and 6-311++G** basis set. Plane-wave cut-off value of 600 Ry was used. The structures of selected oligomers derived from the literature and are shown in Figure S4.[9,10] For each of the oligomers, several possible conformers were generated and their stability both in a vacuum and within the zeolite (vide infra) was tested at CAM-B3LYP and BLYP-D3 level of theory, respectively. We find that while in a vacuum the “trans” type of oligomers is the most stable, the “cis” form is preferred in the zeolite (Table S4). Therefore, for all subsequent calculations we used “cis” type of oligomers.

Vertical electronic excitation energies were determined by using Time-Dependent DFT (TD DFT) as implemented in ADF package[11–14] at the CAM-B3LYP/TZV2P[15] level of theory, which has proven to be accurate for modelling of absorption bands of five-membered heterocyclic compounds[16]. The ability of CAM-B3LYP functional to correctly reproduce exited states of furfuryl alcohol and its derivates was further confirmed by comparing experimentally measured absorption maximum of furfuryl alcohol in water. The experimental value 217 nm[17] is very close to TDDFT computations (220 nm) providing that

a polarisable continuum model (PCM) for water is used, therefore we conclude that CAM-B3LYP functional can be used to resolve main spectral features of UV/Vis absorption of the furfuryl alcohol oligomerization reaction products.

To account for a flexibility of the UV/Vis-active compounds and confinement arising from the zeolite network, we followed the same procedure as Hemelsoet *et al.*[18]. Different conformations of both neutral and charged oligomers entrapped in zeolite pores were generated by ab-initio Molecular Dynamics (MD) simulations. As model system of the environment for both the neutral and protonated oligomers, silicalite-1 and H-ZSM-5 with a Si/Al ratio of 95 were used, respectively. The Al atom was introduced in the zeolite network by replacing one Si atom located at a T12 position. DFT calculations were performed within the CP2K software and GPW method[19] by using a DZVP MOLOPT basis set, GTH pseudopotentials, and BLYP functional[20,21] with the -D3 dispersion correction scheme of Grimme[22]. MD simulations were performed in the canonical (NVT) ensemble at a temperature 298K with a time step of 0.5 fs. A Nosé-Hoover thermostat of length three was used to control the temperature. Prior each MD simulation, the unit cell of entrapped oligomers was optimized and then kept fixed during the MD run. The unit cell dimensions used for each simulation are summarized in Table S5. The system was initially allowed to equilibrate for 1 ps, which was followed by a 5 ps production run. The time-averaged spectra were obtained by taking 50 different snapshots (every 200 steps), on which separate TD DFT calculations were performed. During TD DFT simulations, the zeolite structure was not taken into account due to enormous computational cost this would require. Finally, the averaged spectrum from all snapshots was computed.

Section S5. Supplementary videos

Supplementary videos 1-2:

wide-field movie of fluorescent molecules diffusing through the sinusoidal channels over a b-oriented zeolite film. Trajectories (lines) and localizations (circles) are overlaid in post-processing. Green circles mark localizations with a width (standard deviation of the Gaussian fit function) deviating less than 30% from the expected 128 nm width, while localizations with a larger deviation are marked red. Temporal resolution: 30 ms per frame. Playback at 15% real-time. In supplemental video 1, a zoom-in of a region with a high density of mobile and hybrid trajectories is shown. Supplemental video 2 displays the same time lapse without zoom.

Supplementary video 3:

wide-field movie of fluorescent molecules diffusing through the straight channels over an a-oriented zeolite film. Trajectories (lines) and localizations (circles) are overlaid in post-processing. Green circles mark localizations with a width (standard deviation of the Gaussian fit function) deviating less than 30% from the expected 128 nm width, while localizations with a larger deviation are marked red. Temporal resolution: 30 ms per frame. Playback at 15% real-time.

Supplementary video 4:

wide-field movie of fluorescent molecules diffusing through the sinusoidal channels over a b-oriented, hierarchical zeolite film. Trajectories (lines) and localizations (circles) are overlaid in post-processing. Green circles mark localizations with a width (standard deviation of the Gaussian fit function) deviating less than 30% from the expected 128 nm width, while localizations with a larger deviation are marked red. Temporal resolution: 30 ms per frame. Playback at 15% real-time

Supplementary video 5:

wide-field movie of fluorescent molecules diffusing through the straight channels over an a-oriented, hierarchical zeolite film. Trajectories (lines) and localizations (circles) are overlaid in post-processing. Green circles mark localizations with a width (standard deviation of the Gaussian fit function) deviating less than 30% from the expected 128 nm width, while localizations with a larger deviation are marked red. Temporal resolution: 30 ms per frame. Playback at 15% real-time.

Section S6. Supplementary Figures S1-S22

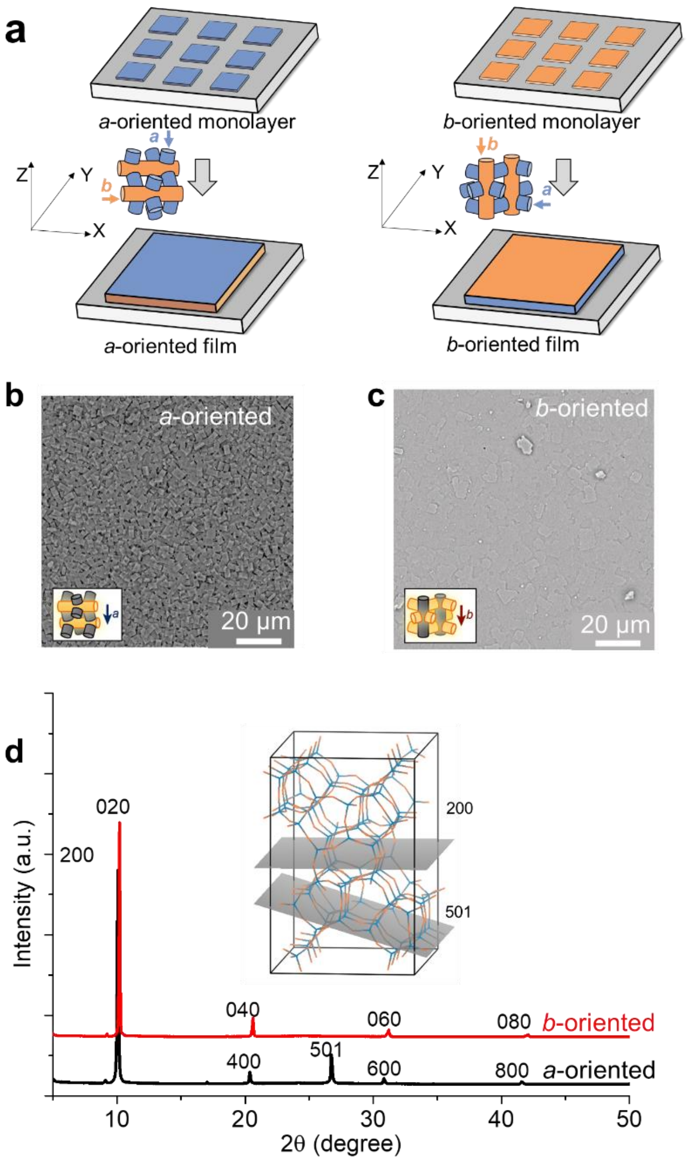

Figure S1. Growth of the oriented zeolite films. a) Illustration of the growth procedures of oriented zeolite films from corresponding oriented zeolite monolayers. b-c) Scanning electron microscope (SEM) images of b) *a*-oriented and c) *b*-oriented zeolite ZSM-5 films grown in the secondary growth media solutions with Si/Al = 125. d) X-ray diffraction patterns of *a*-oriented and *b*-oriented zeolite thin films. The *a*-oriented film shows strong (200), (400), (600) and (800) diffraction peaks as well as a (501) diffraction, which are all consistent with [100] orientation of the crystal. The *b*-oriented film shows (020), (040), (060) and (080) diffractions, consistent with a [010] orientation.

The zeolite films were grown from the same gel solution, so the difference in the *a*-oriented and *b*-oriented films would be solely in their orientations. The *a*-oriented zeolite films are with the sinusoidal and straight channels being perpendicular (*z*-axis) and parallel (*xy*-plane) to the substrates, respectively. Similarly, the *b*-oriented zeolite films are with the straight and sinusoidal channels being perpendicular (*z*-axis) and parallel (*xy*-plane) to the substrates, respectively. Therefore, the channels in the *z*-axis are with the identical sizes and geometries, while those in the *xy*-plane are with the identical sizes and geometries. Due to the polarization of the laser and uniform orientation of the films, solely the molecules align in the channels in the *xy*-plane would be excited by the 560 nm laser. This allows us to exclusively investigate the diffusion behaviour of molecules in the channels with the same size and geometry in the *xy*-plane.

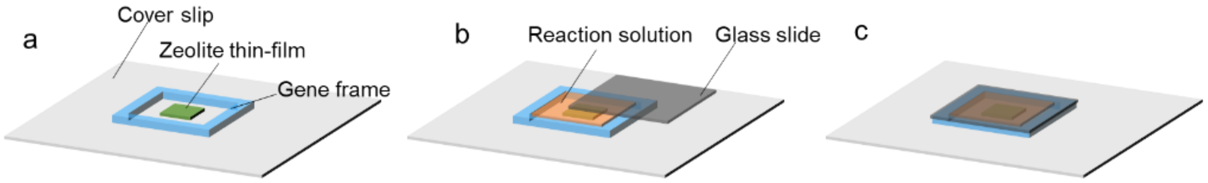

Figure S2. Reaction cell for single-molecule fluorescence (SMF) microscopy experiments. a) A zeolite ZSM-5 film loaded on the coverslip adhered to the Gene Frame. b) The reaction solution of furfuryl alcohol (FFA) diluted with 1,4-dioxane was dropped into the Gene Frame with optimized concentrations (Table S1). c) The reaction cell is closed with a glass slide adhered to the gene frame.

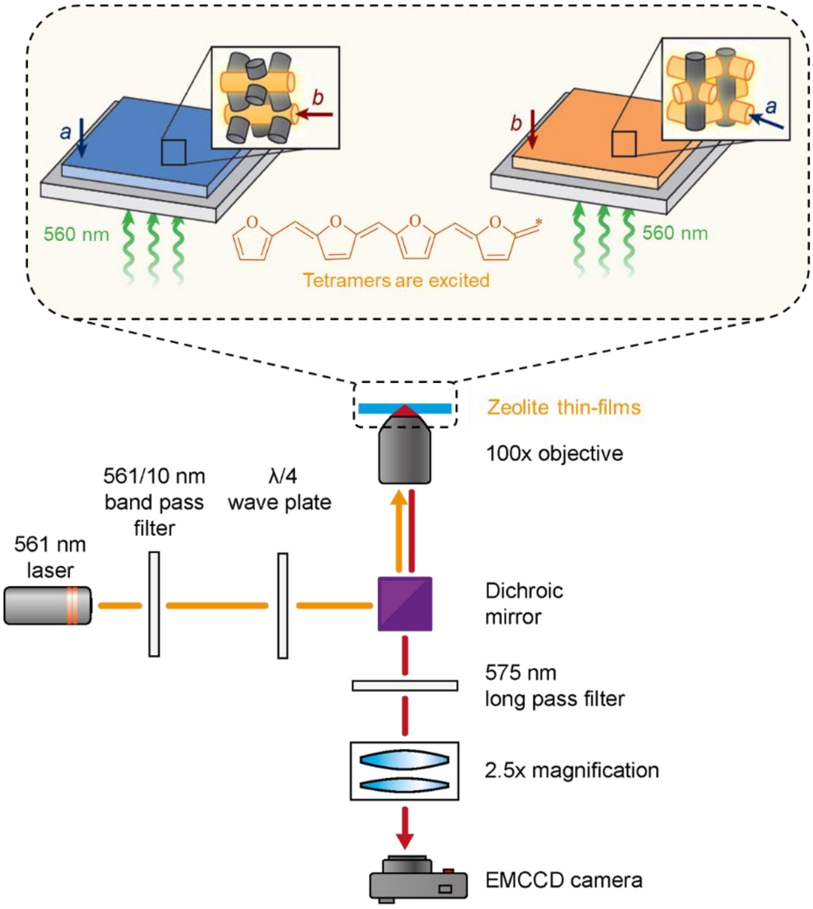

Figure S3. Schematic representation of the inverted epifluorescence widefield fluorescence microscope. The output of a 560-nm diode-pumped solid-state laser was spectrally filtered, the polarization changed to circular, and then focused on the centre of the back-focal plane of an oil-immersion objective with numerical aperture 1.49. This created an illumination spot with 25 μm diameter on the zeolite film. Fluorescence from the sample was collected by the same objective, spectrally separated from reflected excitation light with a dichroic mirror and a 575-nm long-pass filter and imaged on an electron-multiplying charge-coupled device (EMCCD) with an overall magnification of 250×. The tetramers from the furfuryl alcohol oligomerization are excited by the 560 nm laser, and their movement within the straight and sinusoidal zeolite channels were tracked by the single-molecule fluorescence localization microscopy. The results are listed in Table S2. Additionally, the stochastic single turnover dynamics were quantified.

Oligomerization of furfuryl alcohol catalysed over Brønsted acid sites in zeolites

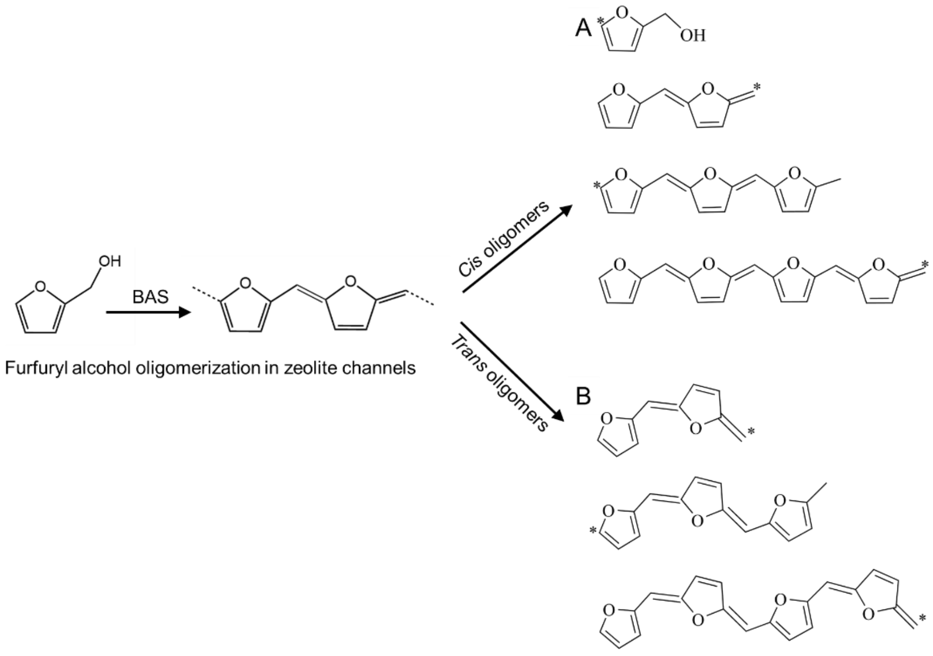

Figure S4. Schematic representation of possible oligomerization products of furfuryl alcohol catalysed over Brønsted acid sites in zeolite channels. Two major conformers were considered, *i.e.*, the *cis* (A) and *trans* (B) and conformation. Within the zeolite, the *cis* conformation is more stable. Both neutral and cationic species were considered; the protonation site is indicated by an asterisk. The absorption maxima of these oligomers were calculated using the time dependent density functional theory.

The absorption spectrum and emission properties of the FFA oligomerization reaction products

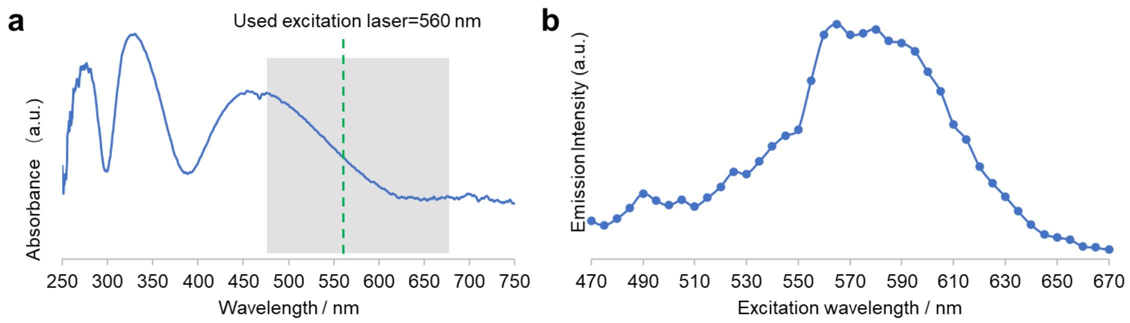

Figure S5. The absorption spectrum and emission properties of the FFA oligomerization reaction products. a) Absorption spectrum of the FFA oligomerization reaction products, determined with UV/Vis diffuse-reflectance spectroscopy on a representative ZSM-5 zeolite thin film. b) The intensity of fluorescence signal as a function of excitation wavelength detected from the same film. The emission is detected at a wavelength longer than the corresponding excitation wavelength by 20 nm. The range of excitation wavelength measured is highlighted in panel a. The absorption spectrum of the reaction products of furfuryl alcohol (FFA) oligomerization (Figure S5a) shows three distinct absorption bands cantered at 260 nm, 330 nm, and 460 nm. The strongest fluorescence is observed when exciting between 560 and 600 nm (Figure S5b). We, therefore, chose 560 nm as the excitation wavelength for our single-molecule tracking experiments.

Time-dependent quantification of the stochastic single turnover dynamics

To determine the catalytic behaviour of our zeolite thin films, we counted the number of new trajectories starting per unit of time, assuming that each start represents the formation of a new product molecule.[32] We found that the turnover rate increased in the initial stages of the experiment for both channel orientations and approached a constant value after approximately 2 hours of reaction (Figures 6a–b). A previous study of large ZSM-5 zeolite crystals showed a continuous increase of stochastic single turnovers as a function of time, which was attributed to the long diffusion path of ~20 μm in the large crystals.[32] However, our zeolite thin films have a short diffusion path (~800 nm), so FFA molecules can readily diffuse into the micropores of the zeolites and reach a steady-state concentration distribution within approximately 2 hours as shown in Figure S6c. Additionally, a sharp decrease in turnover rate was observed in the first 2 min of each measurement (not shown), indicating photobleaching of the molecules. To avoid this effect, 5 min of laser illumination was performed before each diffusion measurement.

The different reactivity in the straight and sinusoidal channels can affect the mobility of the molecular probes. As a result of a different reactivity, the fluorescent oligomers can have a different length, which directly affects the probe's mobility. We argue that the effect of the reactivity on the obtained diffusion constants is relatively small if we make two assumptions: a higher reactivity would result in a longer oligomer and this would lead to a lower diffusion constant. If the observed difference in probe diffusivity between the straight and sinusoidal pores were due to longer probes as a result of a higher reactivity, we expect a negative correlation between the reactivity and diffusivity. That says, a lower diffusion coefficient would be obtained for the straight channels. However, we find a positive correlation between the reactivity (Figure S6) and diffusivity (Table S2) in the parent samples. Therefore, we conclude that the different reactivity is not a dominant factor in the probe diffusion.

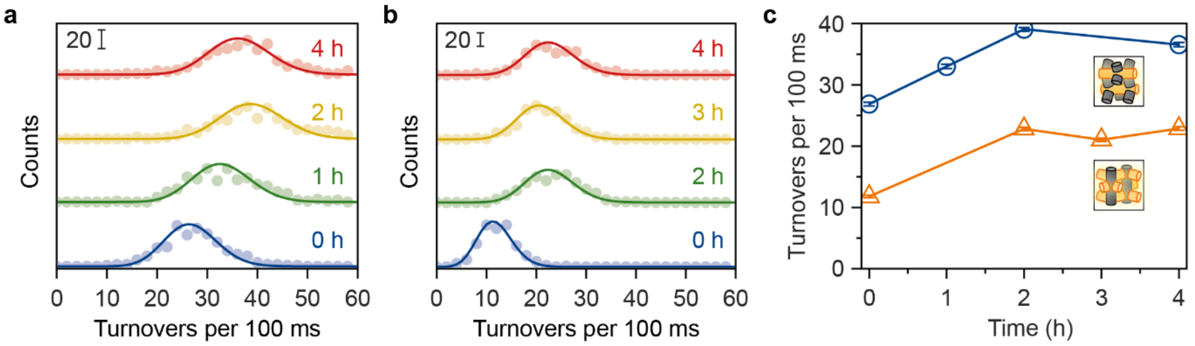

Figure S6. Single turnover statistics of the FFA oligomerization reaction. a–b) Stacked histogram of the total number of detected turnovers per 100 ms in a) an *a*-oriented and b) a *b*-oriented zeolite film measured over 30 seconds after various reaction times. The filled circles are the experimental data, and the solid line is a fit of the Poisson distribution of the experimental data. c) The average turnover rates measured from panels a) and b), denoted with symbols ○ and △, respectively. The error bars represent the standard error in the fit of the Poisson mean and are smaller than the symbols.

Time-dependent density functional theory calculated absorption spectra of furfuryl alcohol oligomers

Time-dependent density functional theory (TD-DFT) calculations were performed to identify the UV-Vis absorption bands of furfuryl alcohol oligomers, and the results are summarized in Table S3 and Figures S7-8. First, the absorption spectra of both protonated and neutral forms in the vacuum were considered. The absorption maximum of the protonated species was always at longer wavelengths than the neutral form. As shown in Figure S7, the absorption bands of the neutral oligomers increased progressively in wavelength from 209 nm (monomers) to 475 nm (tetramers) and the bands of the pronated oligomers from 219 nm (monomers) to 574 nm (tetramers). From comparison with the experimental data (Figure S5a), it is evident that the absorption band around 460 nm corresponds to conjugated species, namely trimeric and tetrameric species. This is a guideline, because absorption bands of confined molecules can shift with respect to their unconfined equivalents as has been demonstrated by Hemelsoet *et al*.[18]

The influence of the zeolitic framework on the oligomers trapped in the pores of the MFI zeolite was examined using ab-initio MD simulations. The results are compiled in Figure S8. A correlation between the number of oligomer units and the position of absorption maxima is found, which is in agreement with reports of other five-membered heterocyclic compounds, such as thiophenes.[10] The calculated absorption maximum of monomeric species is between 212 and 277 nm for the neutral and protonated form, respectively, and does match the experimental absorption band cantered at 260 nm. The experimentally observed band around 330 nm is closest to the calculated absorption of dimer species (377–404 nm for neutral and protonated form). The band around 460 nm overlaps with calculated absorption maximum of trimeric species cantered at 453 nm and 499 nm in its respectively neutral and protonated form. The only species that absorbs above 500 nm is the highly conjugated tetramer with a calculated absorption maximum at 544 and 614 nm for its neutral and protonated form. As we shown in Figure S8, the absorption maximum of the oligomers increases ca. 100 nm with the addition of an extra monomer. Therefore, we expected that the absorption for slightly larger oligomers, *e.g.,* pentamer, would be with an absorption maxima > 644 and 714 for the neutral and protonated forms, respectively. The experimental results, however, show that the highest absorption observed for this reaction over zeolites is *ca.* 620 nm. This unambiguously suggest that the largest and dominant stable species formed during the furfuryl alcohol oligomerization that can be excited by a 560 nm laser, is the furfuryl alcohol tetramer. However, as shown in Table S3, the adsorption peaks are further broadened by presence of different

conformations. As the result, calculated standard deviation for the dynamic spectra is up to 32 nm. Additionally, in our model we assume that oligomers are located in the straight zeolite channels. In a real system, they are formed in both straight and sinusoidal channels, which will result into further broadening of adsorption maxima. Thus, it cannot be excluded that using 560 nm laser we have excited also other species such as trimer. The tetramers, however, would be the most efficiently excited species as they show adsorption maximum close to 560 nm.

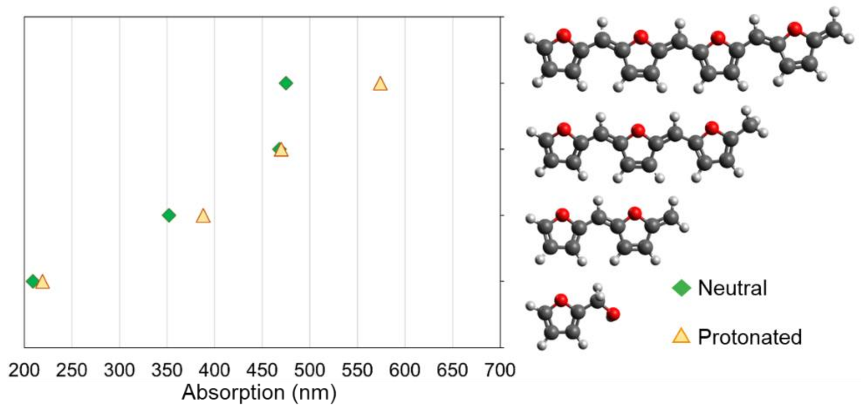

Figure S7. TD-DFT calculated absorption maxima for oligomers with optimized geometries. The detailed parameters for calculation and the absorption maxima are summarized in Tables S3-S5.

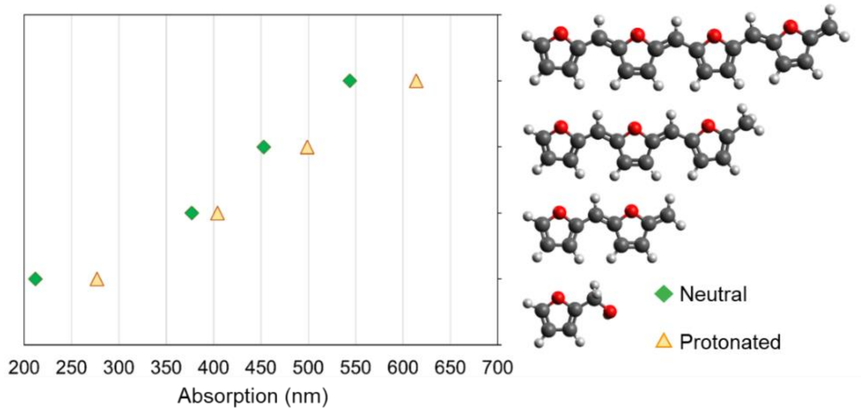

Figure S8. TD-DFT calculated absorption maxima for oligomers trapped in zeolite channels. The detailed parameters for calculation and the absorption maxima are summarized in Tables S3-S5.

Determination of pixel jump and blinking gap

We use a tracking algorithm to identify single-molecule localizations that are likely the same molecule based on their vicinity in space and time. These localizations are grouped into what we call a "trajectory". In the grouping process, two input values are important: the "pixel jump" and the "blinking gap" (Figure S9). The blinking gap accounts for the possibility that the fluorescence of a single molecule "blinks", *i.e.* sometimes turns off for a few tens of ms.[33] The blinking gap is the maximum number of dark frames by which we allow a trajectory to be interrupted. Similarly, the pixel jump specifies the maximum spatial separation between two consecutive localizations in a trajectory.

Finding the optimal set of cut-off values is challenging, and even the most optimal set can limit the range of diffusion coefficients that can be reliably extracted from the experiments. If the pixel jump value is too small, the algorithm does not recognize that two consecutive localizations originate from the same molecule when they are further spaced apart from the pixel jump (Figure S9a). The trajectory is falsely cut, and our motion analysis will measure a reduced average diffusion constant. Instead, if the pixel jump is too large, we will connect localizations of different molecules that are in each other's vicinity, which will increase the measured average diffusion constant in our analysis. Therefore, we aim at a pixel jump value that minimizes the probability of either scenario.

We have manually determined the optimal pixel jump and blinking gap by careful inspection of the trajectories overlaid with the recorded movies. Where the tracking algorithm cannot distinguish between localizations from different molecules, we humans sometimes can make an educated guess about the localizations that belong to the same molecule, for instance by considering the brightness of the localizations. By making such educated guesses, we select the largest value of the pixel jump and blinking gap that does not connect localizations of different molecules. We use these values in our analysis, which are given in Table S7.

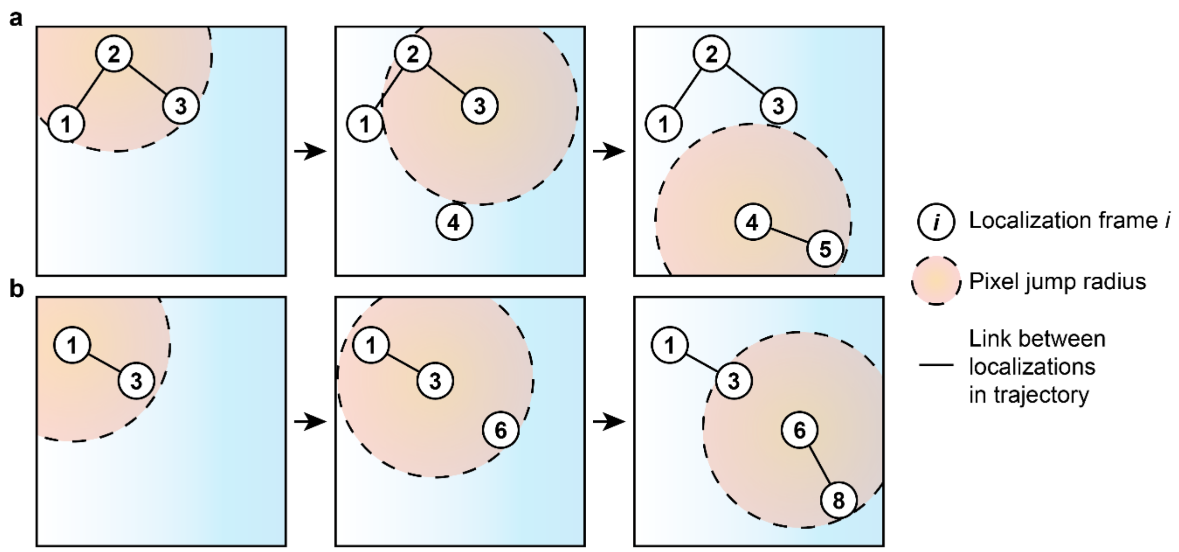

Figure S9. Examples of the grouping process by the tracking algorithm. a) The first example demonstrates the grouping process for a molecule that does not blink, and we only have to consider the pixel jump. When the pixel jump is shorter than the displacement between two subsequent localizations, such as between localization two and three, it is recognized by the algorithm. However, if the displacement is larger than the pixel jump, which happens between localization three and four, the displacement is not recognized. The trajectory is then falsely cut and we obtain two measured trajectories. b) In the second example, the molecule blinks and we also have to consider the blinking gap. Depending on its value, the algorithm still recognizes a displacement even though the molecule is off for one or more frames. The blinking gap is the maximum number of dark frames allowed and its value is one in this example. Thus, a pair of localizations with displacement shorter than the pixel jump will be recognized, if the number of dark frames between the localizations does not exceed one. For instance, between localizations one and three, the molecule blinks for one frame and the displacement is recognized. However, between localization three and six, the molecule is dark for two frames, which is longer than the blinking gap of one. The trajectory is falsely cut even though the displacement is smaller than the pixel jump, and we obtain two measured trajectories.

Modelling of the effect of the pixel jump on the measured diffusion constant

Depending on the pixel jump value, our tracking algorithm may tend to underestimate the diffusion constant of the fastest molecules in the sample. These molecules would show large displacements, which the tracking algorithm fails to recognize. When this happens frequently, the probability decreases that all localizations of a single molecule are grouped in the same trajectory. This probability is an important parameter, which we coin the “trajectory recognition success probability”. A probability close to one means that the algorithm is able to recognize the molecules, while a probability close to zero shows that it often misses displacements and is not able to group all localizations of a molecule in the same trajectory. We want the algorithm to recognize the full trajectory, so a probability close to one is desired. In this section, we will model the trajectory recognition success probability to estimate whether we can reliably measure the diffusion constant of the fastest molecules in the sample. We do not consider the false grouping of different molecules into a trajectory, because we have manually selected values of the pixel jump and the blinking gap at which this should not occur.

To model the trajectory recognition success probability, we need to compute the probability that the displacements of every single step in the trajectory are recognized by the tracking algorithm. We start by modelling the recognition success probability of a single step in the trajectory, which allows us to compute the recognition success probability of the full trajectory. First, we consider a molecule that is continuously fluorescent and does not blink. This means that all steps in the trajectory span one frame. We assume 2-dimensional Brownian motion with a displacement probability in *x* and *y* given by a normal distribution with a standard deviation of $\sqrt{2Dt}$, with D the diffusion coefficient and t the delay time.[34] We assume that the error in the localizations is normally distributed with a standard deviation of σ. The recognition success probability of a single step in the trajectory p in the case of no blinking[35] is given by

$$p_1 = 1 - e^{\frac{-\Delta r_{max}^2}{\mathrm{MSD}}} \tag{1}$$

where Δr_{max} is the pixel jump and MSD is the mean squared displacement. The subscript for p_1 indicates that the displacement of this step spans one frame, which corresponds to a molecule that does not blink, and the MSD depends on the frame time Δt as

$$\mathrm{MSD} = 4D\Delta t + 4\sigma^2. \tag{2}$$

The probability that a full trajectory is recognized by the tracking algorithm is given by

$$P = p_1^{N-1} \tag{3}$$

with N the length of the trajectory in frames. This situation is depicted in Figure S10a.

Blinking affects the probability that a single-molecule trajectory is properly identified. During periods when the molecule is off, it cannot be localized, and localizations are missing. These so-called "localization gaps" in the trajectory result in steps that span more than one frame. We can generalize equations (1) and (2) for steps that span n frames

$$p_n = 1 - e^{\frac{-\Delta r_{max}^2}{\text{MSD}}} \tag{4}$$

with

$$\text{MSD} = 4Dn\Delta t + 4\sigma^2. \tag{5}$$

To compute the trajectory recognition success probability, we multiply all values of p weighted in the exponent by their occurrence in the trajectory. The probability that a full trajectory is recognized by the tracking algorithm becomes

$$P = p_1^h p_2^k p_3^l \cdots \quad \text{with} \quad 1h + 2k + 3l + \cdots = N - 1. \tag{6}$$

The indices h, k, l are the number of times a step spanning $n =$ 1, 2, 3 frames occurring in a trajectory, respectively. The length of the trajectory N includes frames in which the molecule is non-fluorescent. This relation is depicted for a single trajectory in Figure S10b. The step recognition success probability p_n as a function of the diffusion constant is shown in Figure S10c. Depending on the blinking gap, we get a different number of factors in equation (6). The maximum number of n is always one frame larger than the blinking gap. The average values of h, k, l depend on the blinking statistics of our molecules and the length of the trajectory N. These values can be fractional as they are computed from the mean over many trajectories.

We do not know the blinking statistics of the molecules. Therefore, we estimate the average h, k, l values from the statistical distribution of localization gaps in the experimental data. The length of these localization gaps is per definition limited by the blinking gap that was used in the tracking. Therefore, we can only approximate the true blinking statistical distribution from the experimental data. Localization gaps that are longer than the blinking gap are not measured. We cannot estimate these gaps from the experimental data and do not consider these in our analysis. This is not a problem for the diffusion

constant estimation, because these long localization gaps do not affect the measured diffusion constant. When a step in a trajectory spans over more frames than the blinking gap allows, the trajectory is falsely cut. The cutting is done irrespective of the magnitude of the displacement and therefore does not lead to an underestimation of the diffusion constant. Altogether, we can safely omit localization gaps larger than the blinking gap in our statistical analysis.

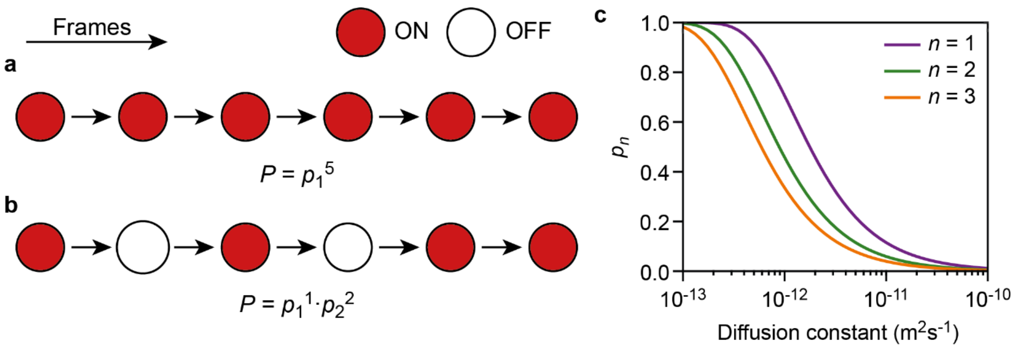

Figure S10. Trajectory recognition success probability. a–b) Schematic representation of a trajectory with a length ($\mathbf{N}$) of six frames without a) and with b) blinking. The trajectory recognition success probability $\mathbf{P}$ is given below each trajectory. c) The recognition success probability of a single displacement $\mathbf{p_n}$ given as function of the diffusion constant. The localization error $\boldsymbol{\sigma}$ was fixed at 10 nm, pixel jump $\Delta\mathbf{r_{max}}$ at 384 nm (6 pixels), and the frame time $\Delta\mathbf{t}$ at 30 ms.

We find that the probability that a full trajectory of a molecule is recognized by the tracking algorithm strongly depends on its diffusion constant and the length of the trajectory. We can plot this as a probability map if we assume a constant localization error, pixel jump, and frame time (Figure S11a). We find a very low recognition probability for fast molecules ($\mathrm{D} = 10^{-11} - 10^{-12}$ m^2s^{-1}), near-perfect detection for slow molecules ($\mathrm{D} = 10^{-14} - 10^{-13}$ m^2s^{-1}), and a transition region between $\mathrm{D} = 10^{-12} - 10^{-13}$ m^2s^{-1}. The transition region is important because it shows the fastest molecules that can be recognized by the tracking algorithm. We see that trajectories with a shorter length have a higher probability to be successfully recognized by the algorithm. The transition region moves to a lower diffusion constant when the pixel jump is decreased from six to two pixels (Figure S11a–b). We can understand this intuitive result if we again realize that a shorter pixel jump limits the maximum displacement we can measure. This leads to a lower probability to successfully recognize the step, therefore a decreased probability to

recognize the full trajectory. In the end, this means that the diffusion constant of the fastest molecules we can accurately estimate is lower at a shorter pixel jump.

Blinking affects the trajectory recognition success probability as well, and we find a broadening of the transition region due to blinking (Figures 11a and 11c). This broadening is a result of many different combinations of localization gaps that can occur in a trajectory within the same statistical distribution. We also find that the transition region moves to a slightly lower diffusion constant. The p decreases for a larger localization gap: $p_1 > p_2 > p_3 > \cdots$, because the molecule can have a larger displacement, as it has more time to travel between the detections (Figure S11c). Therefore, it has a lower probability to have a displacement that is shorter than the pixel jump, which moves the transition region to a lower diffusion constant.

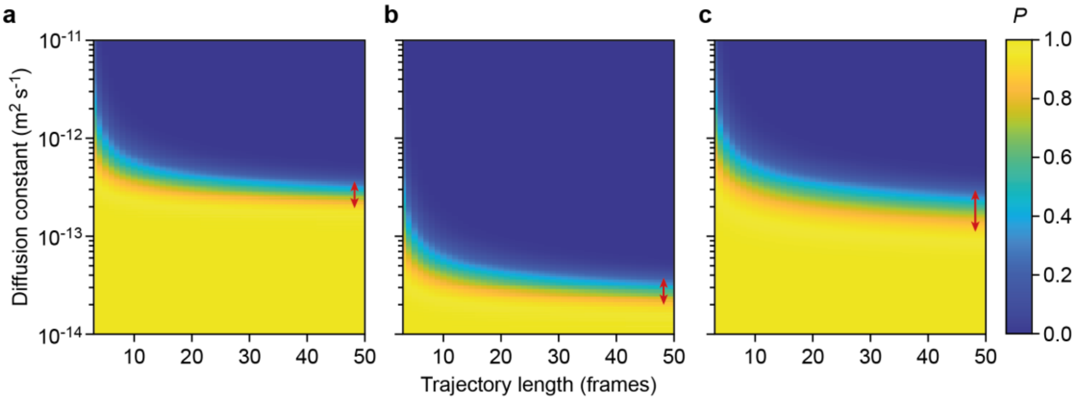

Figure S11. The impact of the pixel jump and blinking characteristics on the trajectory recognition success probability. Maps of the trajectory recognition success probability $\mathbf{P}$ as a function of the diffusion constant $\mathbf{D}$ and trajectory length $\mathbf{N}$. The red arrows indicate the position and width of the transition region for a trajectory length of 48 frames. a–b) Probability maps without blinking with a fixed pixel jump of a) 384 nm (6 pixels) and b) 128 nm (2 pixels). We see a very low recognition probability for fast molecules ($\mathbf{D}$ = 10^{-11} – 10^{-12} m^2s^{-1}), near perfect detection for slow molecules ($\mathbf{D}$ = 10^{-14} – 10^{-13} m^2s^{-1}), and a transition region between $\mathbf{D}$ = 10^{-12} – 10^{-13} m^2s^{-1}. A smaller pixel jump moves the transition region to a lower diffusion coefficient. c) Probability map with a constant pixel jump of 384 nm (six pixels) and blinking statistics of the mobile trajectories in the *a*-oriented parent zeolite thin film. The transition region broadens and moves to a slightly lower diffusion constant as a result of blinking (see *e.g.,* red arrows). The localization error $\boldsymbol{\sigma}$ was fixed at 10 nm and the frame time $\Delta\mathbf{t}$ at 30 ms.

Validating the pixel jump

To quantitatively compare the experimentally obtained diffusion constants, we must be sure that we do not underestimate the diffusion constant of the fastest molecules. If we do not underestimate the diffusion constant of the molecules, we call the pixel jump “good”—otherwise it is “too short”. We can investigate whether the pixel jump is good by comparison of the experimental trajectories with the trajectory recognition success probability map. Every trajectory is a point on the probability map, and from the distribution of trajectories, we can learn whether the pixel jump is good or too short. The problem is that we do not know the true diffusion constant D and trajectory length N of a measured trajectory; however, we can learn how the trajectory behaves near the transition region by looking at simulated trajectories with a known D and N.

First, we need to learn how the distribution of trajectories on the probability map looks like if the pixel jump is good. To this end, we simulate trajectories with a diffusion constant and statistical distribution of localization gaps representative for the experiments, and we call these the “true” trajectories. We assume two-dimensional Brownian motion as described above. The simulated trajectories were first converted into localizations. These localizations were linked back to new trajectories, which we call the “measured” trajectories. The linking was done with the same pixel jump and blinking gap as was used in the experiment. We simulate true trajectories with a length of 40 frames and diffusion constant of 10^{-13} m^2s^{-1} and overlay this with the probability map of P (Figure S12a). According to our analytical model, the simulation parameters result in trajectories with $P \approx 0.9$. We see that most measured trajectories span 40 frames, like the true trajectories, which confirms that the pixel jump is good. A few measured trajectories were shorter than the true trajectories, because of the ~ 0.1 probability that the true trajectory is not successfully recognized at $P \approx 0.9$.

Now that we know how the distribution of trajectories looks if the pixel jump is good, we simulate trajectories for which the pixel jump is too short. We simulate trajectories with a diffusion constant slightly higher ($5 \cdot 10^{-13}$ m^2s^{-1}) and far higher than the transition region (10^{-12} m^2s^{-1}), and overlay the resulting “measured” trajectories with their probability map (Figure S12b–c). The pixel jump is too short according to our model, since the simulated parameters would result in $P < 0.1$ for both simulations. Because the longest displacements are not included in the measured trajectories, we expect that these trajectories become shorter and have a lower diffusion constant than the true trajectories. We indeed find this trend

in our simulation results. Overall, this leads to many trajectories in the blue-colored region of the probability maps (with $P < 0.2$), which is in contrast to the simulation with a good pixel jump where we didn't find any trajectories in this region. We will use this characteristic to estimate whether the experimentally measured trajectories are linked by a pixel jump that is too short.

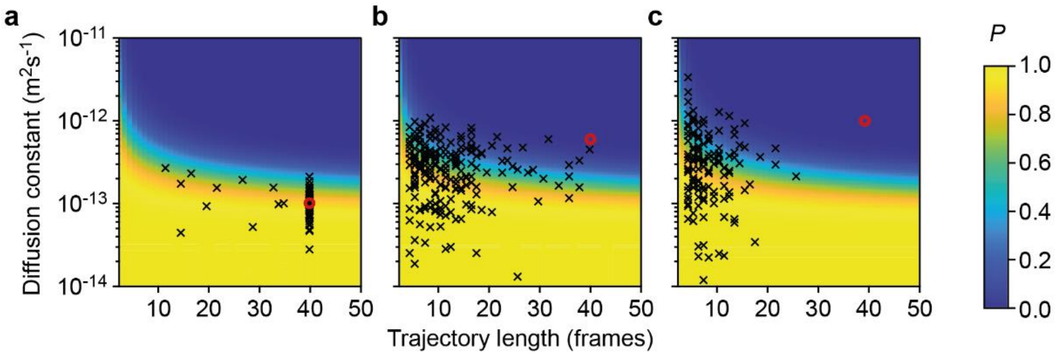

Figure S12. Validation of the trajectory recognition success probability maps with simulated trajectories. Maps of the trajectory recognition success probability $\mathbf{P}$ as a function of the diffusion coefficient $\boldsymbol{D}$ and the trajectory length $\mathbf{N}$ is given for each set of simulated trajectories. Overlaid black crosses are the "measured" simulated two-dimensional trajectories originating from 100 "true" simulated trajectories with a diffusion constant of a) 10^{-13}, b) $5{\cdot}10^{-13}$ c) 10^{-12} m^2s^{-1}. The true trajectories were simulated spanning 40 frames and with a statistical distribution of localization gaps of the mobile trajectories in the a-oriented parent zeolite film. Linking into measured trajectories was performed with a pixel jump of six pixels and a blinking gap of three frames. The diffusion coefficient of the measured trajectories was obtained with an MSD analysis fitting the first 25% or at least three points. The red dots mark the input parameters of the true trajectories.

We compare the experimentally obtained trajectories with their probability map of P (Figures S13a–d) and do not find strong indications that the pixel jump is too short. Indeed, all trajectories are in or below the transition region with a $P > 0.2$. If, on the other hand, we take a smaller pixel jump value of 2 pixels, we find clear signatures that this affects our estimates of diffusion constants (Figures S13e–g). Here, a significant portion of the trajectories is in the $P < 0.2$ domain, which indicates that the pixel jump is indeed too short. Altogether, this analysis demonstrates a proper selection of the pixel jump that does not introduce errors in the estimated diffusion constant.

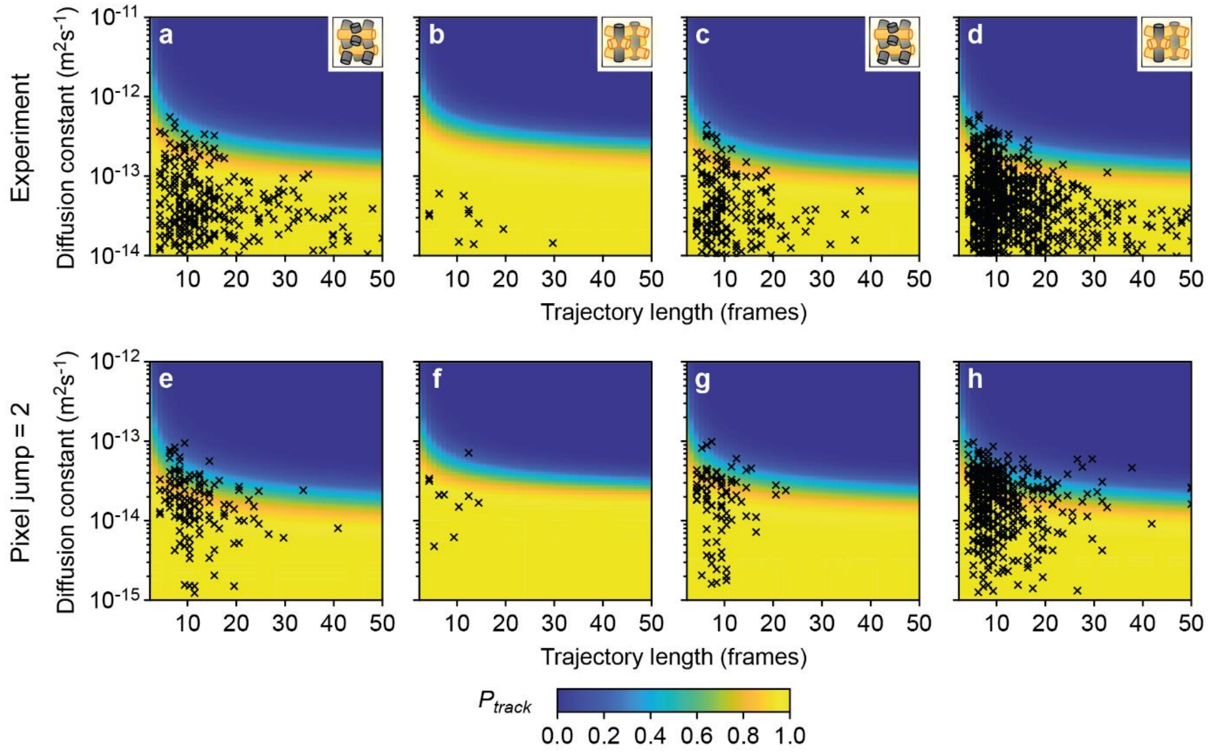

Figure S13. Trajectory recognition success probability maps for experimentally obtained trajectories. Map of the probability to detect the true trajectory **P** as a function of the diffusion coefficient **D** and the trajectory length **N**. Overlaid black crosses are the experimental mobile trajectories linked with a pixel jump of a–b) 6, c–d) 5 and e–h) 2 pixels. The mobile trajectories were obtained from parent a,e) *a*-oriented and b,f) *b*-oriented as well as hierarchical c,g) *a*-oriented and d,h) *b*-oriented zeolite ZSM-5 films. The statistical distribution of localization gaps of the corresponding trajectories is used in the model. Black crosses represent individual trajectories, where the diffusion coefficient was obtained with an MSD analysis fitting the first 25% or at least three points. The value of the y-axis for (e–h) is an order of magnitude lower than that for (a–d).

Validation of the diffusion heterogeneity observed by single-molecule tracking

As discussed in the main text, linear fits of the MSD curves of each trajectory in a sample produce a continuous range of diffusion coefficients produce a continuous range of diffusion coefficients spanning eight orders of magnitude (Figure 2a), which reflects the strong heterogeneity within theoretically identical straight zeolite channels. It should be noted that the imprecision in localization also contributes to the span of diffusion coefficients, particularly at small diffusion constants. Nevertheless, Figure S14 shows a distribution of “displacements”, which is largely independent of the delay time. Closer inspection reveals a small shoulder at long displacements that increases with the delay time (inset of Figure S14), which is attributed to molecular diffusion. Therefore, these results collectively suggest the existence of strong diffusion heterogeneity, and thus simply calculating the average diffusivity of the tracked molecules would ignore this important information. We performed MSD analysis after trajectory classification on individual trajectories and the results are shown in Figures 3a–b and Figures S22a–b. We find a spread of more than two orders of magnitude in the diffusion coefficient, which is due to heterogeneity in the underlying motion and imprecision of the diffusion coefficient estimation. The distribution of diffusion coefficients is skewed and contains outliers—also to negative values. In these conditions, the median of the distribution is preferred to compare the populations. Because the distribution is not normal, the standard error of the median could not be computed. Although correlation in the displacements could lead to a large spread in the estimated diffusion coefficients[28], our simulation shows that the variation is about 1 order of magnitude. Therefore, the up to 6 orders of magnitude variation of diffusion coefficients for trajectories in the same type of trajectories reflects the true heterogeneity of diffusivity in zeolite channels.

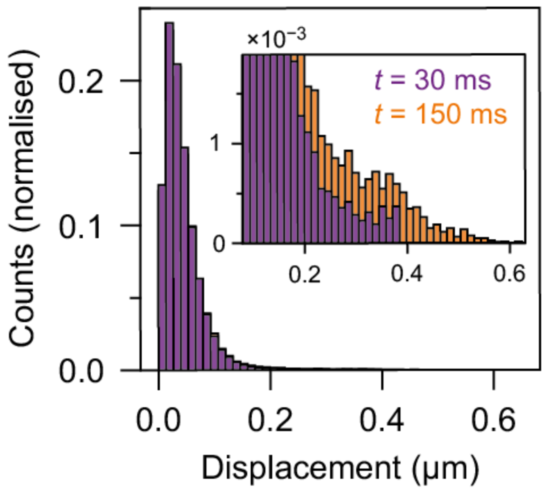

Figure S14. Histogram of the displacements of all trajectories within straight channels. The data was obtained from the *a*-oriented ZSM-5 film over a delay time ***t*** of 30 ms (1 frame, purple) and 150 ms (5 frames, orange). The inset zooms in on longer displacements.

Classification of trajectories

The procedures for classification of trajectories are shown in Figure S15, with a detailed list of corresponding trajectory properties listed in Table S6. We build on the trajectory properties and hierarchical decision tree described in Hendriks and Meirer et al.[24] to classify the trajectories into mobile, hybrid and immobile populations. Inspired by this previous work in which trajectories were classified using a machine learning approach,[24] we separated the trajectories using a rationally designed decision tree after a detailed analysis of the influence of trajectory properties on the classification. Only trajectories that have at least five displacements (**a**) were considered to ensure sufficient displacements for mean squared displacement analysis. The diameter of the smallest enclosing circle (**b**) is a good property to classify immobile trajectories. The localizations of the immobile trajectories are scattered around a fixed point, *i.e.* the true location of the immobile molecule, due to imprecision in the localization of the molecules. The diameter of the area over which the localization is spread is related to the localization error and can be described by the diameter of the smallest enclosing circle. Our threshold value is 128 nm, which is larger than in previous work[24] due to the large localization error of dim molecules. However, some immobile trajectories even have a diameter larger than 128 nm. A closer look at these residual trajectories showed that the shape of immobile and mobile/hybrid trajectories was different, with a circular shape for immobile trajectories and a more linear shape for the other trajectories. Therefore, an elongation (**h**) threshold of 30% was used to take the residue of immobile trajectories apart. To separate the mobile trajectories from the hybrid ones, the entropy (**i**) property was found to be the most effective parameter. This parameter quantifies the randomness in the spatial distribution of localizations. Hybrid trajectories have domains with mobile and immobile displacements, while the mobile trajectories have merely mobile displacements. This yields a different distribution of trajectory coordinates and thus a different randomness. We classified the mobile and hybrid trajectories in the final split of the decision tree. Mobile trajectories had an entropy smaller than 0.08, while the remaining trajectories were classified as hybrid trajectories.

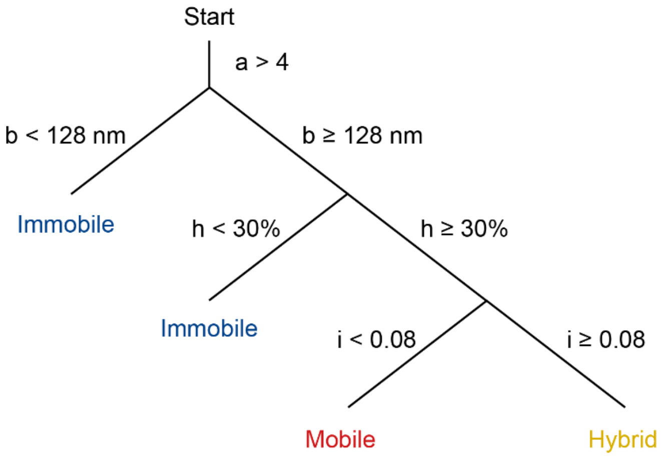

Figure S15. The decision tree used for the classification of trajectories. Tracks were grouped into immobile, hybrid, and mobile categories. The descriptions of the properties of a, b, h, and i can be found in Table S6.

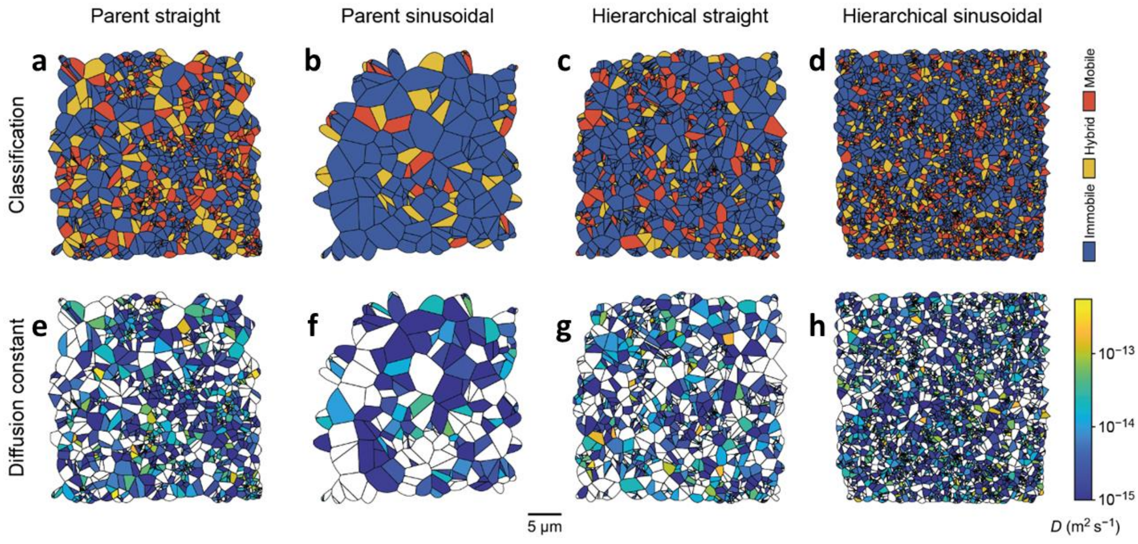

Figure S16. Spatial map of the classified trajectories and corresponding diffusion constants. a-d) are the spatial maps of the classified trajectories in all zeolite films. e-h) are the spatial maps of corresponding diffusion constants. The color-coding of the populations is immobile (blue), hybrid (yellow), and mobile (red). Trajectories with a negative slope of the MSD are shown in white. The area assigned to a trajectory is the Voronoi polygon of the centre of mass of the trajectory. The diffusion coefficients are calculated from MSD curves from individual molecules using free diffusion model.

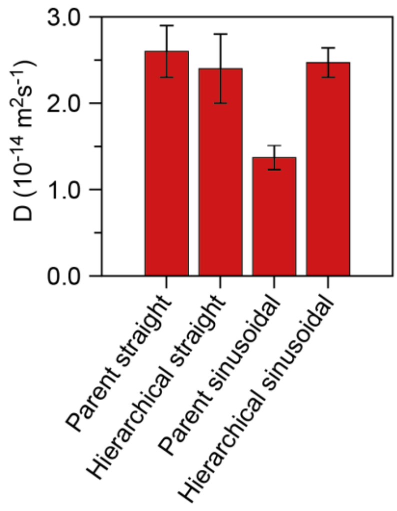

Figure S 17. The diffusion coefficients of the mobile trajectories obtained from the population ensemble MSD fit. The error bars indicate the standard error of the fit.

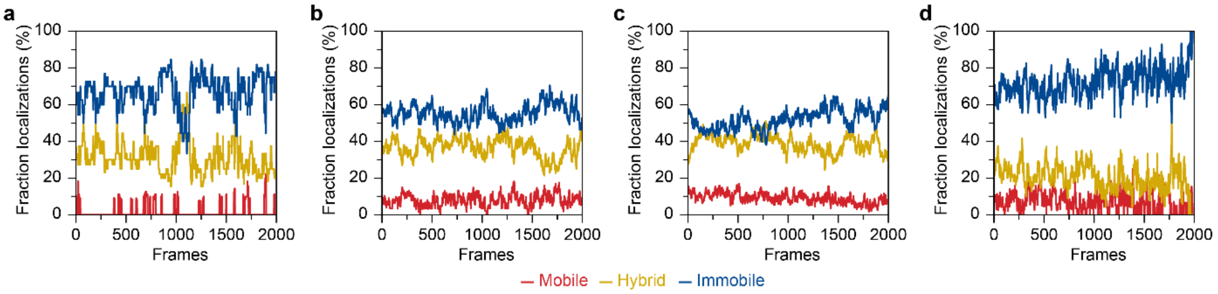

Figure S 18. The fraction of localizations per mobile, hybrid, and immobile population per movie frame. The time evolution of the fraction of localizations per population is shown: a) parent sinusoidal, b) parent straight, c) hierarchical sinusoidal, and d) hierarchical straight. The fraction of localizations for each population was used to calculate the effective diffusion coefficient.

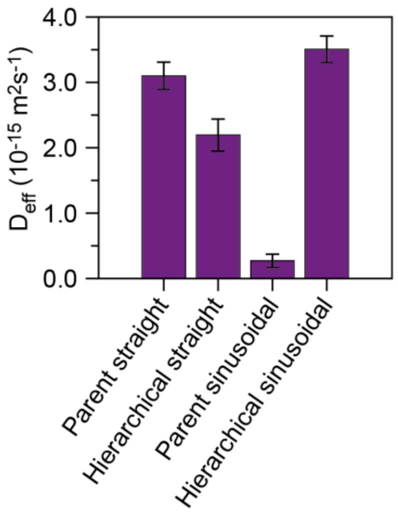

Figure S 19. The effective diffusion coefficients obtained from the population ensemble MSD fit.

The error bars indicate the standard error.

Morphology analysis of zeolite films after the introduction of secondary porosity

Several approaches, including dealumination (*i.e.,* steaming and acid leaching) and desilication (*i.e.,* alkaline leaching) have been widely applied to produce meso- and macropores.[36,37] However, these approaches also change the chemical composition and the related spatial distribution of silicon and aluminium within zeolites and thereof their acidities. To exclusively study the effect of the introduction of meso- and macroporosity on the diffusion properties, we have applied a recently developed method, *i.e.,* NH_4F etching, to introduce secondary porosity by equally removing silicon and aluminium.[4] focused ion beam-scanning electron microscopy (FIB-SEM) analysis proved the successful introduction of secondary porosity to the zeolite films. Figure S20 shows that the intercrystallite domains grown from secondary growth are severally etched, demonstrating that these domains are more vulnerable to a fluoride attack. Similarly, Qin et al. reported that small intergrown domains on the surface of zeolite ZSM-5 crystals were removed.[38] Moreover, the formation of macropores was also observed at the surface of zeolite crystals, as shown in the high-resolution scanning electron microscopy (SEM) images in **Figure** S20. A closer look has found small discrete domains with sizes of ~50 nm at around the crystal edges, most likely demonstrating the formation of mesopores in the zeolite crystals. To clearly illustrate the formation of mesopores, we also have attempted to study the morphology of the cross-sections using FIB-SEM.[39] As shown in Figures S20c–d, big intercrystallite gaps were generated after NH_4F etching, further confirming the vulnerability of the intergrown structures towards this treatment. Some mesoporous features were also observed in the edge of the zeolite ZSM-5 crystals, which we believe are the mesopores generated by NH_4F etching. Additionally, using transmission electron tomography, Qin *et al.* also demonstrated that the secondary pore networks produced by NH_4F etching of zeolite ZSM-5 are ordered and well-aligned.[4] The formation of these nano-sized domains was attributed to the oriented attachment of nanocrystal precursors. Indeed, Rimer et al. demonstrated that the growth of silicalite-1 crystals involves the growth of silica both classical pathways based on the attachment of silica molecules followed by a disorder-to-order rearrangement and nonclassical pathways where metastable silica nanoparticles are the primary growth units.[40] Nonetheless, well crystalized zeolite ZSM-5 crystals are eventually formed from oriented nanocrystal units, which can be dissolved by NH_4F leading to the formation of mosaic, oriented nanopores.[4] As the same etching approach was utilized, we postulated that the secondary pore network in the zeolite films also possess a similar arrangement being ordered and well-aligned.

To confirm the formation of mesopores and further highlight morphological changes to the film morphology, atomic force microscopy (AFM) measurements were performed on the *b*-oriented zeolite films prior and post etching, as shown in Figure S21. We selected *b*-oriented zeolite films as a representative as the material is known for its flat surface, which is suitable for AFM measurements. The parent sample (Figure S21a) shows typical zeolite crystal step edges of 1.3 ± 0.4 nm high, as calculated from the line profiles shown in Figure S21b. This value is equal to the height of the building unit of MFI, *i.e.,* a pentasil chain.[41] Furthermore, a surface roughness, represented by a calculated RMS, of 2.4 and 3.0 nm can be found for the full zoom-in micrographs. If we only consider one crystal plane, a roughness of 0.3 – 0.5 nm was found. This altogether suggests the formation of a perfect *b*-oriented zeolite film that is free of other pore networks.

The micrographs of the etched sample (Figure S21c) confirm the successful introduction of secondary pore networks. Interestingly, the surface around the formed pores seems to be rougher and displays a granular structure as seen in the zoom-ins. The overall surface roughness of the samples increases to significant higher values (7.0 and 12.0 nm, excluding the cracks) and the crystal step edges are hard to be found, although still visible at certain locations, like in the micrograph with smaller Z-contrast (dashed blue box). This micrograph also highlights the large number of secondary pores found at the crystals. From the displayed micrographs, all visible secondary pores were measured over the largest cross section and represented in the approximated pore size distribution in Figure S21d, indicating a median pore size of 112 nm. It should be noted that this is the pore size found at the surface, as the AFM is only to measure top-down and the cone-like tip shape prevents entering the pores if they are smaller than the tip apex.

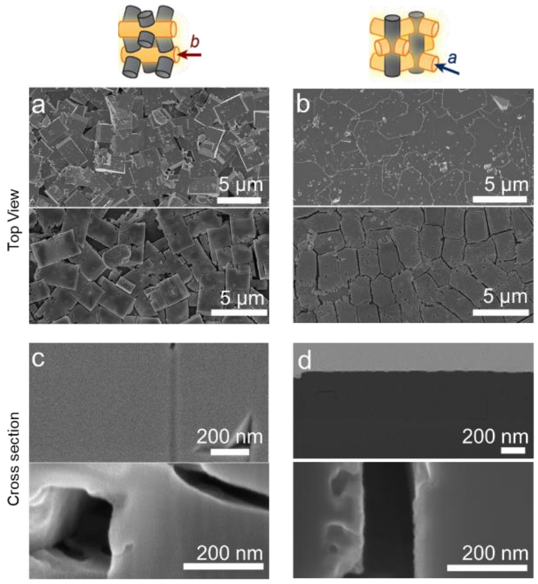

Figure S 20. The morphologies of zeolite films with secondary pores. Scanning electron microscope (SEM) images of the (a, c) *a*-oriented and (b, d) *b*-oriented zeolite ZSM-5 films before (top) and after (bottom) 40 wt% NH_4F etching.

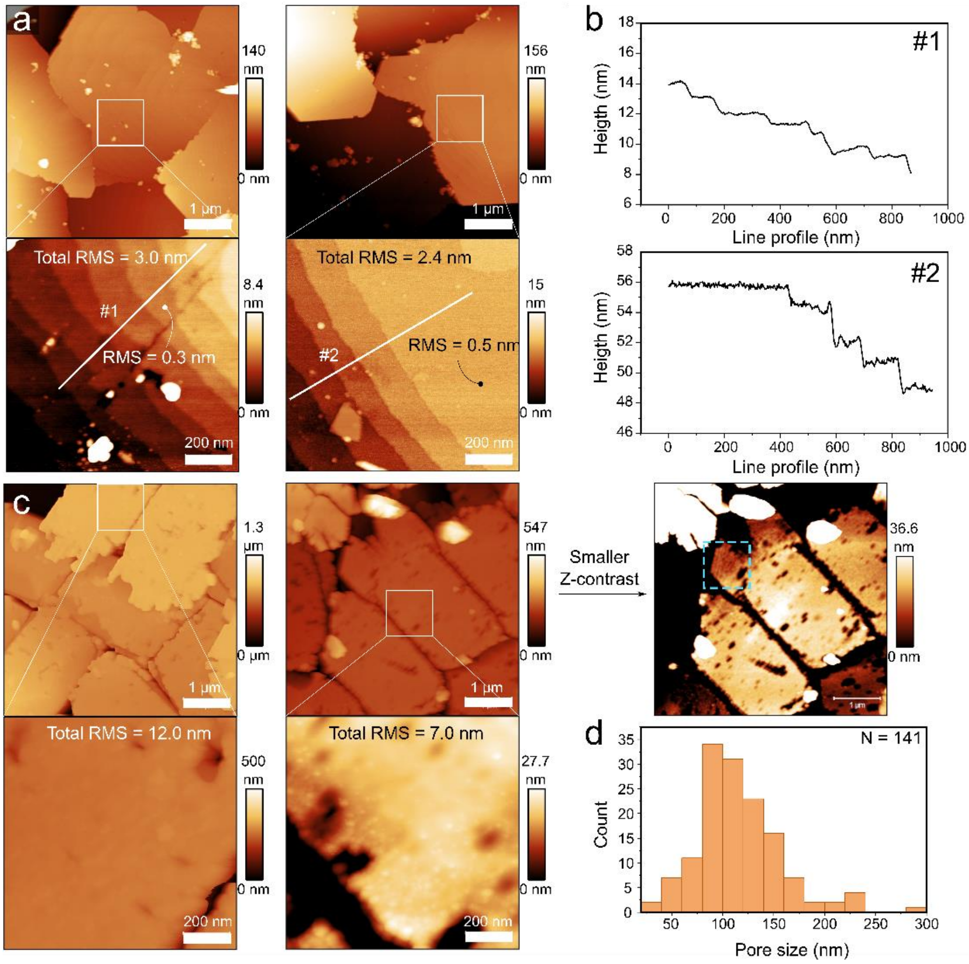

Figure S 21. Surface topology of the *b*-oriented zeolite film with secondary pores. Atomic force microscope (AFM) height micrographs of (a) parent *b*-oriented zeolite ZSM-5 films on two different spots. Zoom-in micrographs are given as well, with their position indicated by the white boxes. (b) Line profiles are shown to highlight the zeolite crystal steps, which are 1.3 ± 0.4 nm high. (c) AFM height micrographs and zoom-in of the *b*-oriented zeolite ZSM-5 film after etching with 40 wt% NH_4F, showing the rougher film surface and formation of mesopores, (d) which are found to have a median pore size of 112 nm.

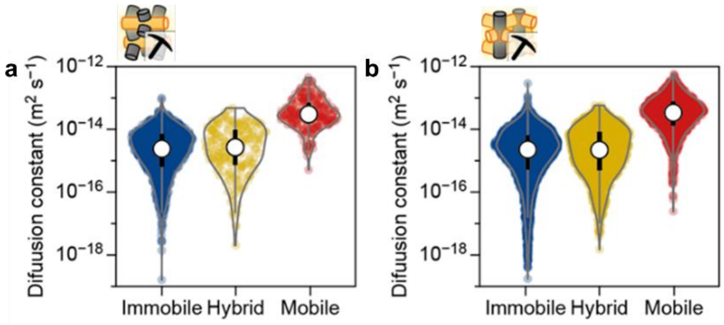

Figure S 22. Violin plot of diffusion coefficients for the classified trajectories in zeolite films with secondary pore networks. The results were obtained via MSD analysis on individual trajectories in the a) straight and b) sinusoidal zeolite channels after the introduction of secondary pore networks. The white dot and the bold black line indicate the median and the first to third quartile of the distribution, respectively. Note that the diffusivity for the straight and sinusoidal zeolite channels are obtained from the *a*-oriented and *b*-oriented ZSM-5 zeolite films, respectively.

Section S7. Supplementary Tables S1-S9

Table S 1. Overview of the optimal concentration of furfuryl alcohol in 1,4-dioxane obtained for the best single-molecule fluorescence measurements.

**Zeolite films**	**Concentrations of Furfuryl Alcohol in 1,4-Dioxane (mmol/L)**	
	***a*-oriented**	***b*-oriented**
Parent	0.001	0.02
Hierarchical	0.01	0.05

Table S 2. Overview of the MSD analysis results for the population and individual trajectory fit. The diffusion coefficient (*D*), localization error (ε), standard error of the fit (σ), and the number of trajectories is given. The median (med.) of the distribution of *D* and ε of the individual trajectories have been rounded to three digits.

**Population**	**Sample**[a]	**Channels tracked**[b]	**Population fit**		**Individual trajectory fit**		**Trajectories**	
			***D* ± σ (m^2/s) x10^{-14}**	**ε ± σ (m) x10^{-8}**	**Med. *D* (m^2/s) x10^{-14}**	**Med. ε (m) x10^{-8}**	**Numbers**	**Fraction (%)**
Mobile	*b*-parent	Sinusoidal	1.38 ± 0.11	4.06 ± 0.11	0.477	4.28	29	11
Mobile	*a*-parent	Straight	2.64 ± 0.24	4.03 ± 0.20	1.73	3.92	476	22
Mobile	*b*-hierar	Sinusoidal	2.51 ± 0.13	4.42 ± 0.09	1.50	4.65	1592	20
Mobile	*a*-hierar	Straight	2.49 ± 0.29	4.47 ± 0.21	1.44	4.42	337	14
Hybrid	*b*-parent	Sinusoidal	0.007 ± 0.009	2.292 ± 0.017	0.00159	3.38	52	19
Hybrid	*a*-parent	Straight	0.267 ± 0.013	2.454 ± 0.019	0.0643	3.25	541	24
Hybrid	*b*-hierar	Sinusoidal	0.31 ± 0.04	2.81 ± 0.05	0.0384	3.19	1761	22
Hybrid	*a*-hierar	Straight	0.21 ± 0.04	3.12 ± 0.05	0.0173	3.27	337	14
Immobile	*b*-parent	Sinusoidal	0.026 ± 0.006	2.694 ± 0.010	0.00131	2.56	188	70
Immobile	*a*-parent	Straight	0.045 ± 0.004	2.527 ± 0.006	0.000177	2.65	1193	54
Immobile	*b*-hierar	Sinusoidal	0.028 ± 0.011	2.253 ± 0.018	0.00368	2.37	4717	58
Immobile	*a*-hierar	Straight	0.012 ± 0.010	2.207 ± 0.018	0.00551	2.22	1760	72

Note:

a. M-parent denotes "parent" zeolite films with preferential "M" orientation before the introduction of secondary pore networks. M-hierar denotes "hierarchical" zeolite films with preferential "M" orientation after the introduction of secondary pore networks.

b. The movement of individual fluorescent molecules within the sinusoidal and straight zeolite channels were tracked in the *b*-oriented and *a*-oriented zeolite films, respectively.

Table S 3. Static (geometry optimized) and dynamic absorption maxima for the neutral and protonated oligomers. Geometries for dynamic spectra were obtained from ab-initio Molecular Dynamics (MD) simulations.

	protonated	λ^{static} (nm)	λ^{MD} (nm)	λ^{MD}_{stdev} (nm)
monomer	no	209	212	7
	yes	219	277	9
dimer	no	342	377	19
	yes	378	404	10
trimer	no	468	453	24
	yes	470	499	16
tetramer	no	475	544	32
	yes	574	614	18

Table S 4. The comparison of relative stabilities in kJ/mol of neutral and protonated furfuryl alcohol oligomers in two different conformations (“cis” and “trans”) in different environments: in vacuo (calculated with CAM-B3LYP) and entrapped in the straight channel of MFI zeolite (calculated with BLYP-D3).

	**Protonated**	**vacuum**		**In zeolite**	
		cis	trans	cis	trans
monomer	Yes	-		-	
	No				
dimer	Yes	15.3	0	1.7	0
	No	13.4	0	0	0.8
trimer	Yes	53.8	0	0	12.8
	No	19.7	0	0	18.3
tetramer	Yes	40.5	0	0	24.8
	No	40.2	0	0	52.1

Table S 5. Summarized unit cell dimensions obtained after cell relaxation of furfuryl alcohol oligomer entrapped in the straight channel of silicalite-1 and HZSM-5 for neutral and cationic form, respectively.

	**Protonated**	***a* (Å)**	***b* (Å)**	***c* (Å)**
monomer	Yes	20.293	20.029	13.482
	No	20.301	19.995	13.467
dimer	Yes	20.303	20.035	13.488
	No	20.314	20.015	13.491
trimer	Yes	20.333	20.033	13.503
	No	20.356	19.991	13.507
tetramer	Yes	20.295	20.071	13.574
	No	20.376	20.165	13.520

Table S 6. List of trajectory properties considered in the decision tree for trajectory classification.

	Parameter name	Parameter description
a	Number of localizations	Number of consecutive single molecule emitters detected.
b	Diameter of the smallest enclosing circle	Diameter of the smallest circle that can be drawn around all the locations in a trajectory.
c	Centre of the smallest enclosing circle	Centre coordinates of the smallest circle that can be drawn around all the locations in a trajectory.
d	Centre of mass based on all points of a trajectory	Mean coordinate of all localizations in a trajectory
e	Distance between ***c*** and ***d***	The distance between the centre of the smallest enclosing circle and the centre of mass based on all points gives an indication of how points are distributed spatially. It is calculated as a percentage of the trajectory's diameter ***b***.
f	Path length	Sum of all consecutive displacements
g	Tortuosity	The Euclidian distance between start and end coordinate divided by the length of the trajectory ***f***.
h	Elongation	Covariance eigen value of the localizations rescaled from 0.5-1 to 0-1
i	Entropy	A statistical measurement of randomness (Shannon's entropy) applied to the distribution of the points within the enclosing square that is defined by the smallest enclosing circle.

Table S 7. Overview of the used tracking parameters for all samples. The values for the different zeolite pore orientations were kept the same.

**Zeolite films**	**Tracking parameters**	
	**Blinking gap**	**Pixel jump**
Parent	3	6
Hierarchical	2	5

Table S 8. Fraction of localizations per population per frame and its standard error of the mean. These values were used for the computation of the effective diffusion constant and differ from the fractions of trajectories per population, which are given in Table S2.

**Sample**	**Channels tracked**	**Fraction mobile ± σ (%)**	**Fraction hybrid ± σ (%)**	**Fraction immobile ± σ (%)**
b-parent	Sinusoidal	1.8 ± 0.7	31.5 ± 0.4	66.70 ± 0.25
a-parent	Straight	8.06 ± 0.25	36.36 ± 0.19	55.48 ± 0.15
b-hierar	Sinusoidal	9.17 ± 0.20	38.91 ± 0.17	51.93 ± 0.16
a-hierar	Straight	7.1 ± 0.4	20.3 ± 0.4	72.62 ± 0.21

Table S 9. Overview of the effective diffusion coefficient ($\boldsymbol{D}_{\text{eff}}$) and standard error (σ) results for the population fit.

**Zeolite films**	**$\boldsymbol{D}_{\text{eff}}$ ± σ (m²/s) x10⁻¹⁵**	
	***a*-oriented**	***b*-oriented**
Parent	3.10 ± 0.21	0.27 ± 0.10
Hierarchical	2.20 ± 0.24	3.51 ± 0.20

References

1. Choi, J., Ghosh, S., King, L., and Tsapatsis, M. (2006). MFI zeolite membranes from a- and randomly oriented monolayers. Adsorption *12*, 339–360.

2. Pham, T.C.T., Kim, H.S., and Yoon, K.B. (2011). Growth of uniformly oriented silica MFI and BEA zeolite films on substrates. Science *334*, 1533–1538.

3. Fu, D., van der Heijden, O., Stanciakova, K., Schmidt, J.E., and Weckhuysen, B.M. (2020). Disentangling reaction processes of zeolites within single-oriented channels. Angew. Chem. Int. Ed. *59*, 15502–15506.

4. Qin, Z., Melinte, G., Gilson, J.-P., Jaber, M., Bozhilov, K., Boullay, P., Mintova, S., Ersen, O., and Valtchev, V. (2016). The mosaic structure of zeolite crystals. Angew. Chem. Int. Ed. *55*, 15049–15052.

5. Kühne, T.D., Iannuzzi, M., Del Ben, M., Rybkin, V.V., Seewald, P., Stein, F., Laino, T., Khaliullin, R.Z., Schütt, O., Schiffmann, F., et al. (2020). CP2K: An electronic structure and molecular dynamics software package - Quickstep: Efficient and accurate electronic structure calculations. J. Chem. Phys. *152*, 194103.

6. Lippert, G., Hutter, J., and Parrinello, M. (1999). The Gaussian and augmented-plane-wave density functional method for ab initio molecular dynamics simulations. Theor. Chem. Acc. *103*, 124–140.

7. Yanai, T., Tew, D.P., and Handy, N.C. (2004). A new hybrid exchange–correlation functional using the Coulomb-attenuating method (CAM-B3LYP). Chem. Phys. Lett. *393*, 51–57.

8. Lehtola, S., Steigemann, C., Oliveira, M.J.T., and Marques, M.A.L. (2018). Recent developments in libxc — A comprehensive library of functionals for density functional theory. SoftwareX *7*, 1–5.

9. Choura, M., Belgacem, N.M., and Gandini, A. (1996). Acid-Catalyzed Polycondensation of Furfuryl Alcohol: Mechanisms of Chromophore Formation and Cross-Linking. Macromolecules *29*, 3839–3850.

10. Roeffaers, M.B.J., De Cremer, G., Libeert, J., Ameloot, R., Dedecker, P., Bons, A.-J., Bückins, M., Martens, J.A., Sels, B.F., De Vos, D.E., et al. (2009). Super-Resolution Reactivity Mapping of Nanostructured Catalyst Particles. Angew. Chem. Int. Ed. *48*, 9285–9289.

11. Velde, G. te, Bickelhaupt, F.M., Baerends, E.J., Guerra, C.F., Gisbergen, S.J.A. van, Snijders, J.G., and Ziegler, T. (2001). Chemistry with ADF. J. Comput. Chem. *22*, 931–967.

12. Amsterdam Modeling Suite Making Computational Chemistry Work For You Softw. Chem. Mater. https://www.scm.com/.

13. van Gisbergen, S.J.A., Snijders, J.G., and Baerends, E.J. (1999). Implementation of time-dependent density functional response equations. Comput. Phys. Commun. *118*, 119–138.

14. van Gisbergen, S.J.A., Kootstra, F., Schipper, P.R.T., Gritsenko, O.V., Snijders, J.G., and Baerends, E.J. (1998). Density-functional-theory response-property calculations with accurate exchange-correlation potentials. Phys. Rev. A *57*, 2556–2571.

15. Van Lenthe, E., and Baerends, E.J. (2003). Optimized Slater-type basis sets for the elements 1-118. J. Comput. Chem. *24*, 1142–1156.

16. Valencia, D., Whiting, G.T., Bulo, R.E., and Weckhuysen, B.M. (2016). Protonated thiophene-based oligomers as formed within zeolites: understanding their electron delocalization and aromaticity. Phys. Chem. Chem. Phys. *18*, 2080–2086.

17. PubChem Hazardous Substances Data Bank (HSDB) : 711. https://pubchem.ncbi.nlm.nih.gov/source/hsdb/711.

18. Hemelsoet, K., Qian, Q., De Meyer, T., De Wispelaere, K., De Sterck, B., Weckhuysen, B.M., Waroquier, M., and Van Speybroeck, V. (2013). Identification of Intermediates in Zeolite-Catalyzed Reactions by In Situ UV/Vis Microspectroscopy and a Complementary Set of Molecular Simulations. Chem. Eur. J. *19*, 16595–16606.

19. VandeVondele, J., Krack, M., Mohamed, F., Parrinello, M., Chassaing, T., and Hutter, J. (2005). Quickstep: Fast and accurate density functional calculations using a mixed Gaussian and plane waves approach. Comput. Phys. Commun. *167*, 103–128.

20. Becke, A.D. (1988). Density-functional exchange-energy approximation with correct asymptotic behavior. Phys. Rev. A *38*, 3098–3100.

21. Lee, C., Yang, W., and Parr, R.G. (1988). Development of the Colle-Salvetti correlation-energy formula into a functional of the electron density. Phys. Rev. B *37*, 785–789.

22. Grimme, S., Antony, J., Ehrlich, S., and Krieg, H. (2010). A consistent and accurate ab initio parametrization of density functional dispersion correction (DFT-D) for the 94 elements H-Pu. J. Chem. Phys. *132*, 154104.

23. Yau, K.W., van Beuningen, S.F.B., Cunha-Ferreira, I., Cloin, B.M.C., van Battum, E.Y., Will, L., Schätzle, P., Tas, R.P., van Krugten, J., Katrukha, E.A., et al. (2014). Microtubule minus-end binding protein CAMSAP2 controls axon specification and dendrite development. Neuron *82*, 1058–1073.

24. Hendriks, F.C., Meirer, F., Kubarev, A.V., Ristanović, Z., Roeffaers, M.B.J., Vogt, E.T.C., Bruijnincx, P.C.A., and Weckhuysen, B.M. (2017). Single-molecule fluorescence microscopy reveals local diffusion coefficients in the pore network of an individual catalyst particle. J. Am. Chem. Soc. *139*, 13632–13635.

25. Berglund, A.J. (2010). Statistics of camera-based single-particle tracking. Phys. Rev. E *82*, 011917.

26. Vestergaard, C.L., Blainey, P.C., and Flyvbjerg, H. (2014). Optimal estimation of diffusion coefficients from single-particle trajectories. Phys. Rev. E *89*, 022726.

27. Michalet, X. (2010). Mean square displacement analysis of single-particle trajectories with localization error: Brownian motion in an isotropic medium. Phys. Rev. E *82*, 041914.

28. Vestergaard, C.L., Pedersen, J.N., Mortensen, K.I., and Flyvbjerg, H. (2015). Estimation of motility parameters from trajectory data. Eur. Phys. J. Spec. Top. *224*, 1151–1168.

29. Kärger, J., and Ruthven, D.M. (2016). Diffusion in nanoporous materials: fundamental principles, insights and challenges. New J. Chem. *40*, 4027–4048.

30. Schneider, D., Kondrashova, D., Valiullin, R., Bunde, A., and Kärger, J. (2015). Mesopore-Promoted Transport in Microporous Materials. Chem. Ing. Tech. *87*, 1794–1809.

31. Taylor, J.R. (1996). An Introduction to Error Analysis: The Study of Uncertainties in Physical Measurements 2nd Edition. (University Science Books).

32. Ristanović, Z., Hofmann, J.P., De Cremer, G., Kubarev, A.V., Rohnke, M., Meirer, F., Hofkens, J., Roeffaers, M.B.J., and Weckhuysen, B.M. (2015). Quantitative 3D fluorescence imaging of single catalytic turnovers reveals spatiotemporal gradients in reactivity of zeolite H-ZSM-5 crystals upon steaming. J. Am. Chem. Soc. *137*, 6559–6568.

33. Vogelsang, J., Kasper, R., Steinhauer, C., Person, B., Heilemann, M., Sauer, M., and Tinnefeld, P. (2008). A Reducing and Oxidizing System Minimizes Photobleaching and Blinking of Fluorescent Dyes. Angew. Chem. Int. Ed. *47*, 5465–5469.

34. Saxton, M.J. (2007). Modeling 2D and 3D Diffusion. In Methods in Membrane Lipids Methods in Molecular Biology™., A. M. Dopico, ed. (Humana Press), pp. 295–321.

35. Schütz, G.J., Schindler, H., and Schmidt, T. (1997). Single-molecule microscopy on model membranes reveals anomalous diffusion. Biophys. J. *73*, 1073–1080.

36. Feliczak-Guzik, A. (2018). Hierarchical zeolites: Synthesis and catalytic properties. Microporous Mesoporous Mater. *259*, 33–45.

37. Donk, S. van, Janssen, A.H., Bitter, J.H., and Jong, K.P. de (2003). Generation, Characterization, and Impact of Mesopores in Zeolite Catalysts. Catal. Rev. *45*, 297–319.

38. Qin, Z., Lakiss, L., Gilson, J.-P., Thomas, K., Goupil, J.-M., Fernandez, C., and Valtchev, V. (2013). Chemical Equilibrium Controlled Etching of MFI-Type Zeolite and Its Influence on Zeolite Structure, Acidity, and Catalytic Activity. Chem. Mater. *25*, 2759–2766.

39. Karwacki, L., de Winter, D.A.M., Aramburo, L.R., Lebbink, M.N., Post, J.A., Drury, M.R., and Weckhuysen, B.M. (2011). Architecture-dependent distribution of mesopores in steamed zeolite crystals as visualized by FIB-SEM tomography. Angew. Chem. Int. Ed. *50*, 1294–1298.

40. Lupulescu, A.I., and Rimer, J.D. (2014). In Situ Imaging of Silicalite-1 Surface Growth Reveals the Mechanism of Crystallization. Science *344*, 729–732.

41. Shete, M., Kumar, M., Kim, D., Rangnekar, N., Xu, D., Topuz, B., Agrawal, K.V., Karapetrova, E., Stottrup, B., Al-Thabaiti, S., et al. (2017). Nanoscale Control of Homoepitaxial Growth on a Two-Dimensional Zeolite. Angew. Chem. Int. Ed. *56*, 535–539.
